# Virulence Determinants and Antimicrobial Profiles of *Pasteurella multocida* Isolated from Cattle and Humans in Egypt

**DOI:** 10.3390/antibiotics10050480

**Published:** 2021-04-22

**Authors:** Mohamed Sabry Abd Elraheam Elsayed, Samah Mahmoud Eldsouky, Tamer Roshdy, Lamia Said, Nahed Thabet, Tamer Allam, A. B. Abeer Mohammed, Ghada M. Nasr, Mohamed S. M. Basiouny, Behairy A. Akl, Maha M. Nader, Al Shaimaa Hasan, Ahmed Salah

**Affiliations:** 1 Department of Bacteriology, Mycology, and Immunology, Faculty of Veterinary Medicine, University of Sadat City, Sadat City, Minufiya 32897, Egypt; 2 Department of Otolaryngology and Head and Neck Surgery, Faculty of Medicine, Benha University, Benha City, Qalyubia 13511, Egypt; samahmahmoed@yahoo.com; 3 Department of Molecular Biology, Genetic Engineering and Biotechnology Research Institute, University of Sadat City, Sadat City, Minufiya 32897, Egypt; tmr_gebri@yahoo.com (T.R.); ahmed.salah@gebri.usc.edu.eg (A.S.); 4 Department of Clinical Pathology, Faculty of Veterinary Medicine, University of Sadat City, Sadat City, Minufiya 32897, Egypt; lamiaasaid@yahoo.com (L.S.); nahedthabet@yahoo.com (N.T.); tamerallam@yahoo.com (T.A.); 5 Department of Microbial Biotechnology, Genetic Engineering and Biotechnology Research Institute, University of Sadat City, Sadat City, Minufiya 32897, Egypt; beromicro@gmail.com; 6 Department of Molecular Diagnostics, Genetic Engineering and Biotechnology Research Institute, University of Sadat City, Sadat City, Minufiya 32897, Egypt; nasr_mi@yahoo.com; 7 Faculty of Biotechnology, Badr University, Badr City, Cairo 19592, Egypt; Mohamed-Salah@buc.edu.eg; 8 Microbiology Department, Faculty of Agriculture, Zagazig University, Zagazig 44519, Egypt; Beharyakl2005@yahoo.com (B.A.A.); mahanaderdiab@gmail.com (M.M.N.); 9 Department of Medical Pharmacology, Faculty of Medicine, South Valley University, Qena 83523, Egypt; DRelshimaa.hassan@med.svu.edu.eg

**Keywords:** *Pasteurella multocida*, molecular capsular typing, virulence factors, antimicrobial resistance, resistance genes

## Abstract

*Pasteurella multocida* is a Gram-negative bacterium that causes drastic infections in cattle and humans. In this study, 55 isolates were recovered from 115 nasal swabs from apparently healthy and diseased cattle and humans in Minufiya and Qalyubia, Egypt. These isolates were confirmed by *kmt1* existence, and molecular classification of the capsular types showed that types B, D, and E represented 23/55 (41.8%), 21/55 (38.1%), and 11/55 (20.0%), respectively. The isolates were screened for five virulence genes with *hgbA*, *hgbB*, and *ptfA* detected in 28/55 (50.9%), 30/55 (54.5%), and 25/55 (45.5%), respectively. We detected 17 capsular and virulence gene combinations with a discriminatory power (DI) of 0.9286; the most prevalent profiles were *dcbF* type D and *dcbF* type D, *hgbA*, *hgbB*, and *ptfA*, which represented 8/55 (14.5%) each. These strains exhibited high ranges of multiple antimicrobial resistance indices; the lowest resistances were against chloramphenicol, ciprofloxacin, amoxicillin/clavulanic acid, and levofloxacin. The macrolide–lincosamide–streptogramin B methylase gene *erm*(Q), with *erm*(42) encoding MLS_B_ monomethyltransferase, *mph*(E) encoding a macrolide efflux pump, and *msr*(E) encoding macrolide-inactivating phosphotransferase were present. The class 1 and 2 integrons and extended-spectrum β-lactamase genes *intl1*, *intl2*, *bla*_CTX-M_, *bla*_CTX-M-1_, and *bla*_TEM_ were detected. It is obvious to state that co-occurrence of resistance genes resulted in multiple drug-resistant phenotypes. The identified isolates were virulent, genetically diverse, and resistant to antimicrobials, highlighting the potential risk to livestock and humans.

## 1. Introduction

*Pasteurella multocida* is a Gram-negative bacterial agent implicated in multi-host infections. These bacteria cause enzootic neonatal calf pneumonia, shipping fever in weaned calves, and hemorrhagic septicemia (HS) in bison, cattle, and buffalo in many regions of Africa, Asia, the Middle East, and southern Europe [1,2]. Human infection with *P. multocida* shows a wide variety of symptoms, from localized infections after scratches and bites of animals to generalized infections such as respiratory tract infections, sepsis, and meningitis [3]. The infection of cattle, causing bovine respiratory disease (BRD), leads to remarkable economic losses for the cattle industry due to increased deaths, high treatment costs, decreased growth rate, and inferior carcass quality [4]. BRD is a multifactorial disease distinguished by the intrusion of bacterial pathogens to the lower respiratory tract, with the predisposing factors being viral co-infection, weaning stress, poor management, diet changes, exaggerated weather changes, transportation, and commingling at auction markets [2]. *P. multocida* infection is characterized by acute respiratory disease accompanied by a high fever, respiratory distress, nasal discharge, polypnea, and death within a few days. Postmortem examination has elucidated extreme congestion of the trachea, lungs, small intestine, and liver [5]. The diagnosis of pasteurellosis in cattle is based on clinical manifestations, postmortem examinations, and traditional bacteriological techniques, which are considered time-consuming and lacking in reliability [6]. The typing of *P. multocida* is based on the capsule and cell envelope lipopolysaccharides. Serological typing has identified 5 capsular serogroups (A, B, D, E, and F) and/or 16 somatic serotypes [7]. Based on the capsular antigens, *P. multocida* strains are differentiated into the following five serogroups: type A, causing fowl cholera and bovine shipping fever; type B, causing hemorrhagic fever in ungulates; type D, causing atrophic rhinitis in swine; type E, an African serotype, infecting cattle and buffalo; and type F, also causing fowl cholera [8,9]. As traditional serological classification is complicated [10], molecular typing techniques have assigned *P. multocida* into five capsular genotypes (A, B, D, E, and F) [11], and eight LPS genotypes (L1–L8) [12]. The pathogenesis of *P. multocida* is based on several virulence factors, such as genes encoding the formation of the capsule, lipopolysaccharide, fimbriae, adhesins, outer-membrane protein toxins, iron acquisition and iron-regulated proteins, sialic acid metabolism, and hyaluronidase [13]. In many parts of the world, the intensive uncontrolled implementation of different types of antimicrobials for prevention and/or treatment of BRD has increased the rate of antimicrobial resistance [2,14,15,16]. The mechanisms of antimicrobial resistance are complex, and continuous bacterial exposure to antimicrobials can select for resistant strains [14], leading to multidrug resistance cassettes that can be shared among various bacterial pathogens by horizontal gene transfer [17]. Based on this, scrutiny of the utilization of antimicrobials has increased so as to lower the emergence of antimicrobial-resistant animal and human pathogens [2].

The methylation of adenine residue A2058 (*Escherichia coli* numbering) on the 23S rRNA is a fundamental mechanism of resistance to macrolides. This modification hinders the attachment of lincosamides, macrolides, and streptogramin B antimicrobial agents, and is controlled by the *erm* genes encoding RNA methylases [18]. Gram-negative bacteria possess many mechanisms for resistance to β-lactam antibiotics, including the production of β-lactamases. As β-lactam antimicrobials are vital for controlling animal and human bacterial infections, the extended-spectrum β-lactamase-producing strains (ESBLs) are of public health importance and cause serious economic losses in animal production [19]. The ESBLs confer their activity through hydrolysis of the β-lactam ring, causing resistance to such β-lactam antimicrobials as penicillins (amoxicillin and ampicillin), cephalosporins (including the third and fourth generations), and monobactams [20]. It has been reported that there are more than 500 types of β-lactamase produced by different bacterial species. β-lactamase-based resistance could be mediated by plasmid or chromosomal expression [21]. The most frequently observed ESBLs are CTX-M and TEM, which belong to ESBL class A; these are horizontally transferable and can be inhibited by clavulanic acid [22].

This study was designed to detect the prevalence of *P. multocida* in apparently healthy and diseased cattle and humans. We performed molecular classification of the capsular types of the gained isolates and detected different virulence genes so as to elucidate the most prevalent genotypic profiles, using a combination of molecular capsular types and virulence genes to detect the relatedness/diversity among the isolates. We also screened phenotypic antimicrobial resistance of the isolates to 14 antimicrobial agents. Moreover, we investigated the existence of macrolide–lincosamide–streptogramin B (MLS_B_) methylases and other macrolide resistance genes and extended-spectrum beta-lactamase genes as well. We then performed correlation matrix analyses and hierarchical clustering to identify associations between the phenotypic and genotypic features, and origins of these strains. Finally, we elucidated the co-occurrence of antimicrobial resistance, the existence of multiple and extensive drug-resistant isolates, and alternative effective antimicrobial agents.

## 2. Results

### 2.1. Origin, Types of Collected Samples, and P. multocida Isolation

A total of 100 nasal swabs were collected from cattle of variable age (10-day-old calves to 24-month-old mature cows) at Minufiya and Qalyubia localities in Egypt. From the screened cases, 48 were apparently healthy and 52 were diseased, showing signs of depression, fever ≥41.5 °C, loss of appetite, nasal discharge, moist cough, and rapid, shallow breathing. Bacterial isolation confirmed that 6/48 (12.5%) apparently healthy cases (five from Minufiya and one from Qalyubia) were infected, as colonies grew in media, while 44/52 (84.6%) diseased cases (40 from Minufiya and 4 from Qalyubia) were positive for bacterial infection. A total of 15 nasopharyngeal swabs were collected from 5 apparently healthy humans (2 from Minufiya and 3 from Qalyubia) and 10 diseased humans (5 from Minufiya and 5 from Qalyubia) suffering from signs of epiglottitis. The human cases were all animal owners or farm workers. Of the diseased cases, three from Qalyubia and two from Minufiya were positive for bacterial isolates, with a rate of 5/15 (33.3%) (Table 1).

### 2.2. Results of Isolation of P. multocida from Different Cases

The isolation results confirmed that 55 out of 115 samples collected (a total rate of 47.8%) were positive for *P. multocida*, distributed as follows: 49/55 (89.1%) isolates from the diseased cattle and human cases, and 6/55 (10.9%) from the apparently healthy cattle, while there were no isolates from the apparently healthy humans. There were significant differences between the isolates from both types of cases (*p* ≤ 0.05, Supplementary Table S1).

### 2.3. Molecular Confirmation of Isolates and Detection of Capsular Types and Virulence Genes

**Figure 1:**
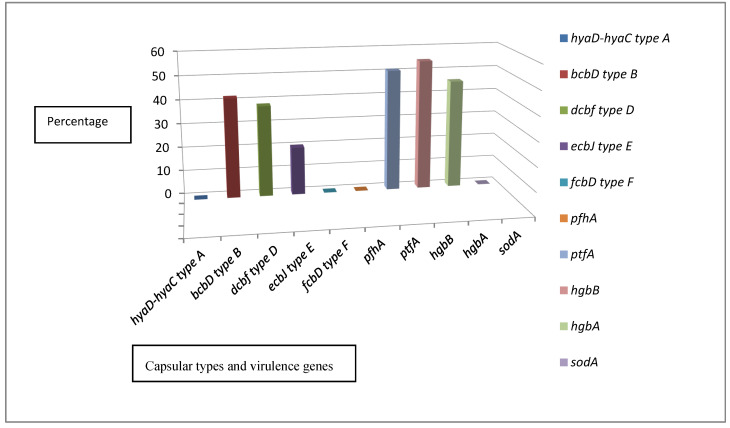
The results of molecular capsular typing and virulence genes.

All 55 isolates were confirmed to be *P. multocida* using primers targeting *kmt1*, a gene for an integral component of the bacterial membrane. The molecular classification of the *P. multocida* capsular types demonstrated that 23/55 (41.8%) of the isolates belonged to type B and were distributed as follows: 18/55 (32.7%) from the diseased cattle, 5/55 (9.09%) from the apparently healthy cattle, and 0/55 (0.0%) from the diseased humans. Moreover, 21/55 (38.1%) of the isolates were type D and were distributed as follows: 15/55 (27.3%) from the diseased cattle, 1/55 (1.8%) from the apparently healthy cattle, and 5/55 (9.09%) from the diseased humans. Furthermore, 11/50 (20%) of the isolates from the diseased cattle were type E. The molecular capsular types A and F were not identified. There was a significant difference between the capsular types (*p* ≤ 0.05). For the screened virulence factors, the genes *hgbA* and *hgbB*, encoding the iron uptake process, were confirmed in 28/55 (50.9%) and 30/55 (54.5%) of the isolates, respectively. The distribution pattern of *hgbA* was as follows: 19/55 (34.5%) from the diseased cattle, 4/55 (7.3%) from the apparently healthy cattle, and 5/55 (9.09%) from the diseased humans. The distribution pattern of *hgbB* was as follows: 20/55 (36.4%) of the isolates from the diseased cattle, 5/55 (9.09%) from the apparently healthy cattle, and 5/55 (9.09%) from the diseased humans. The *ptfA* gene, encoding type IV fimbriae, was confirmed in 25/55 (45.5%) of the isolates and was distributed as follows: 16/55 (29.1%) of the isolates from the diseased cattle, 4/55 (7.3%) from the apparently healthy cattle, and 5/55 (9.09%) from the diseased humans. The genes *sodA*, encoding superoxide dismutase, and *pfhA*, encoding hemagglutinin, were not detected. There was a significant difference between the percentages of virulence genes expressed among groups (*p* ≤ 0.05). Of the isolates from the diseased cattle, 14/55 (25.5%) harbored none of the surveyed virulence genes, while 12/55 (21.8%) of the isolates from the diseased cattle and humans contained 1 virulence gene, 10/55 (18.2%) contained 2 virulence genes, and 8/55 (14.5%) carried 3 virulence genes. Of the isolates from the apparently healthy cases, 1/55 (1.8%) contained 1 virulence gene, 3/55 (5.5%) contained 2 virulence genes, and 2/55 (3.6%) harbored 3 virulence genes. All the isolates from the diseased humans harbored 3 virulence genes (Supplementary Table S2 and Figure 1).

### 2.4. The Obtained Genotypic Profiles Based on Capsular Types and Virulence Genes

**Figure 2:**
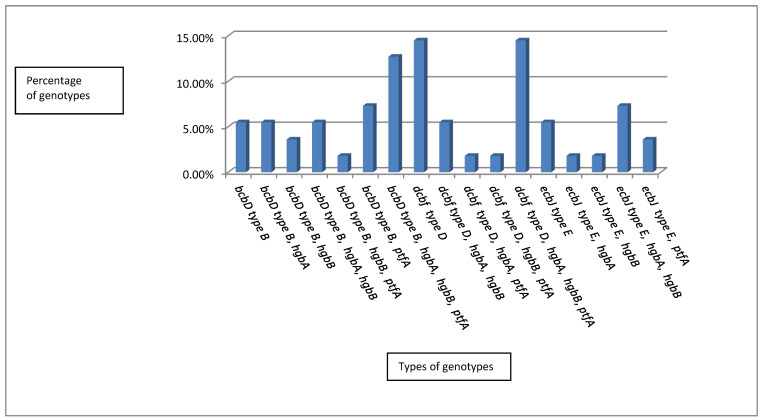
The percentages of the obtained genotypes.

**Figure 3:**
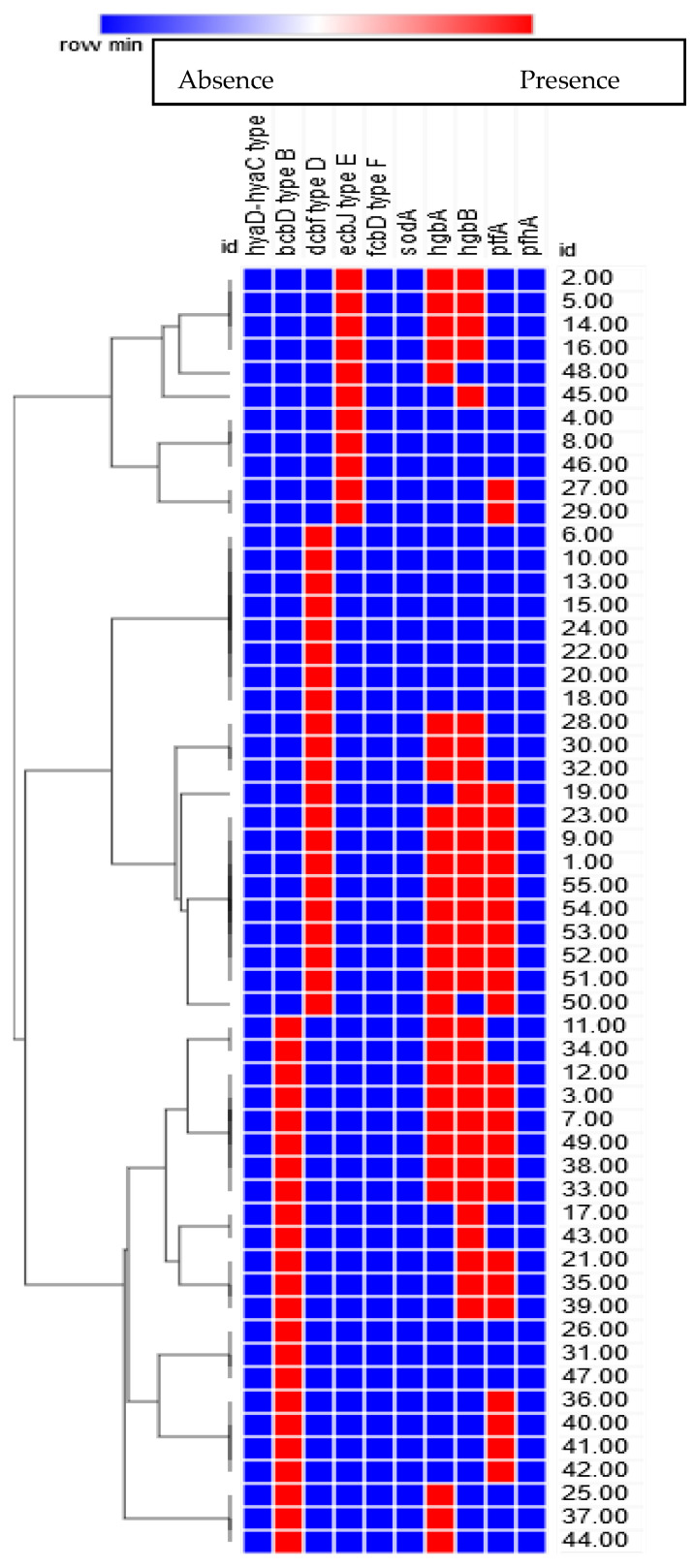
Heatmap and hierarchical clustering of the *P. multocida* isolates to 17 clusters based on the molecular detection of capsular types and virulence genes. The numbers on the right of the heatmap refer to the isolate numbers from 1 to 55.

Based on the molecular classification of capsular type and virulence factor combinations, 17 unique genetic profiles were obtained. The most prevalent profiles were *dcbF* type D and *dcbF* type D/*hgbA*/*hgbB*/*ptfA*, which represented 8/55 (14.5%) each, followed by *bcbD* type B/*hgbA*/*hgbB*/*ptfA*, which was observed in 7/55 (12.7%) of the isolates. Both the genetic profiles *bcbD* type B/*ptfA* and *ecbJ* type E/*hgbA*/*hgbB* were found in 4/55 (7.3%) of the isolates. The genetic profiles *bcbD* type B, *bcbD* typeB/*hgbA*, *bcbD* type B/*hgbA*/*hgbB*, and *ecbJ* type E were identified in 3/55 (5.5%) of the isolates each, while the genetic profiles *bcbD* type B/*hgbB* and *ecbJ* type E/*ptfA* were found in 2/55 (3.6%) of the isolates each. The genetic profiles *bcbD* type B/*hgbB*/*ptfA*, *dcbF* type D/*hgbA*/*ptfA*, *dcbF* type D/*hgbB*/*ptfA*, *ecbJ* type E/*hgbA*, and *ecbJ* type E/*hgbB* were only observed in 1/55 (1.8%) of the isolates each. There was a significant difference between the different genetic profiles (*p* ≤ 0.05, Supplementary Table S3 and Figure 2). The capsular type and virulence factor combinations in the 55 unrelated strains resulted in 17 combination types, which exhibited a high discriminatory power (discriminatory index (DI) of 0.9286; Figure 3).

### 2.5. Phenotypic Antimicrobial Resistance and Multiple Antimicrobial Resistance Index

All 55 *P. multocida* isolates were screened against 14 antimicrobials. The highest resistance was found for the following antimicrobials, in descending order: trimethoprim/sulfamethoxazole (55/55, 100%), oxytetracycline (54/55, 98.2%), danofloxacin (52/55, 94.5%), amikacin (50/55, 90.9%), cefotaxime (50/55, 90.9%), nalidixic acid (48/55, 87.5%), azithromycin (28/55, 50.9%), doxycycline (28/55, 50.9%), erythromycin (28/55, 50.9%), norfloxacin (23/55, 41.8%), ciprofloxacin (16/55, 29.09%), and chloramphenicol (15/55, 27.3%). The lowest resistance was found for the following antimicrobials: amoxicillin/clavulanic acid and levofloxacin, with resistance rates of 10/55 (18.2%) and 7/55 (12.7%), respectively. There was a significant difference between the resistance patterns of all the antimicrobials (*p* ≤ 0.05), and there was no significant difference between the resistance rates against amikacin and cefotaxime, or against chloramphenicol and ciprofloxacin. The multiple antimicrobial resistance (MAR) indices for all the screened isolates ranged from 0.23 to 1 (Supplementary Table S4), with a significant difference between dissimilar MAR indices (*p* ≤ 0.05).

### 2.6. The Existence of Macrolide–Lincosamide–Streptogramin B (MLS_B_) Methylases, Monomethyltransferase, Macrolide Efflux Pump, and Macrolide-Inactivating Phosphotransferase Genes among All the Gained Isolates

**Figure 4:**
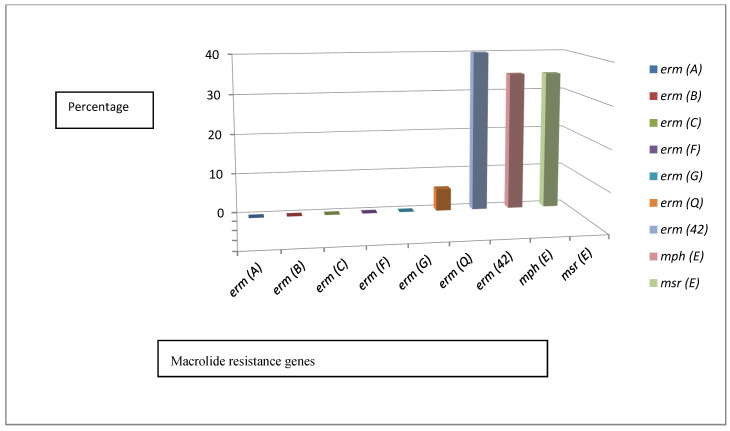
The results of macrolide–lincosamide–streptogramin B (MLS_B_) methylases, monomethyltransferase, macrolide efflux pump, and macrolide-inactivating phosphotransferase genes.

The distribution patterns of macrolide–lincosamide–streptogramin B methylase genes *erm*(A), (B), (C), (F), (G), and (Q), with *erm*(42) encoding MLS_B_ monomethyltransferase, *mph*(E) encoding a macrolide efflux pump, and *msr*(E) encoding macrolide-inactivating phosphotransferase, were screened in all 55 *P. multocida* isolates. The *erm*(Q) gene was found in 3/55 (5.5%) of the isolates, two isolates coded numbers 14 and 48 from the diseased cattle cases with a rate of 3.6%, and one human isolate coded number 55 with a rate of 1.8%. Moreover, *erm*(42) frequency was 22/55 (40%), and 19/55 (34.5%) of the isolates harbored both *mph*(E) and *msr*(E). There was a significant difference between the distribution patterns of the tested genes, with the exception of only *mph*(E) and *msr*(E) (*p* ≤ 0.05). Based on these results, 24/55 (43.6%) of the isolates contained none of these resistance genes, 6/55 (10.9%) of the isolates were resistant to erythromycin, and 5/55 (9.1%) were resistant to azithromycin. Furthermore, 12/55 (21.8%) harbored only a single type of resistance gene, while 9/55 (16.4%) contained 2 different types, 7/55 (12.7%) contained 3 different types, and 3/55 (5.5%) possessed 4 different types of these resistance genes (Supplementary Table S5 and Figure 4).

As predicted for macrolide antimicrobials, there was a high matching rate between the genotype and phenotype as it represented 23/55 (41.8%) and 22/55 (40%) for azithromycin and erythromycin, respectively. For azithromycin, the number of isolates with inactive resistance genes represented 8/55 (14.5%), which was higher than the number of isolates with unexplained resistance phenotypes, which represented 4 (7.2%), and there was no significant difference between them *p* = 0.2205. While the situation for erythromycin was different as one isolate harbored inactive resistance genes, representing 1/55 (1.8%), and was lower than the number of isolates with unexplained resistance phenotypes, which represented 6/55 (10.9%), with a significant difference between them *p* < 0.05 (Supplementary Table S6).

### 2.7. Distribution Patterns of Class 1 and 2 Integrons, Extended-Spectrum β-Lactamase, and Ampicillin-Resistance Genes

**Figure 5:**
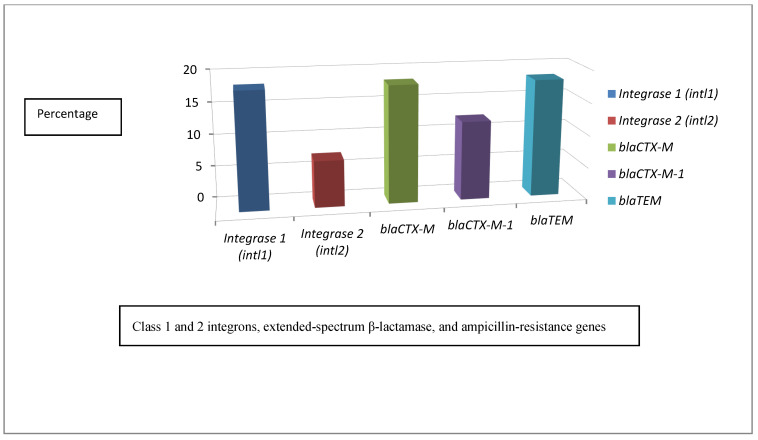
The results of class 1 and 2 integrons, extended-spectrum β-lactamase, and ampicillin-resistance genes.

The presence of class 1 and 2 integrons, extended-spectrum β-lactamase, and ampicillin-resistance genes were investigated in all 55 *P. multocida* isolates. Class 1 integrons were present in 10/55 (18.2%) of the isolates, and were distributed as follows: 1/55 (1.8%) from the apparently healthy cattle, 8/55 (14.5%) from the diseased cattle, and 1/55 (1.8%) from the diseased humans. Class 2 integrons were present in 4/55 (7.3%) of the diseased cattle. The ESBL gene *bla*_CTX-M_ was present in 10/55 (18.2%) of the isolates and was distributed as follows: 2/55 (3.6%) from the apparently healthy cattle, 7/55 (12.7%) from the diseased cattle, and 1/55 (1.8%) from the diseased humans. The *bla*_CTX-M-1_ gene was present in 7/55 (12.7%) of the isolates, and was distributed as follows: 2/55 (3.6%) from the apparently healthy cattle and 5/55 (9.1%) from the diseased cattle. The *bla*_TEM_ gene was present in 10/55 (18.2%) of the isolates, including 2/55 (3.6%) from the apparently healthy cattle, 7/55 (12.7%) from the diseased cattle, and 1/55 (1.8%) from the diseased humans. There was a significant difference between the presence of *bla*_CTX-M-1_ and other ESBLs (*p* ≤ 0.05). A total of 39/55 (70.9%) harbored none of the targeted ESBL genes, including 37/55 (67.3%) sensitive isolates and 2/55 (3.6%) resistant isolates; 4/55 (7.3%) isolates contained a single type of ESBL gene, 5/55 (9.1%) showed 2 different types of ESBL genes, 3/55 (5.5%) possessed 3 different types of ESBL genes, 2/55 (3.6%) had 4 different types of ESBL genes, and 2/55 (3.6%) harbored 5 different types of ESBL genes (Supplementary Table S7 and Figure 5).

### 2.8. Associations between Isolation Source, Strain, and Phenotypic and Genotypic Characteristics

**Figure 6:**
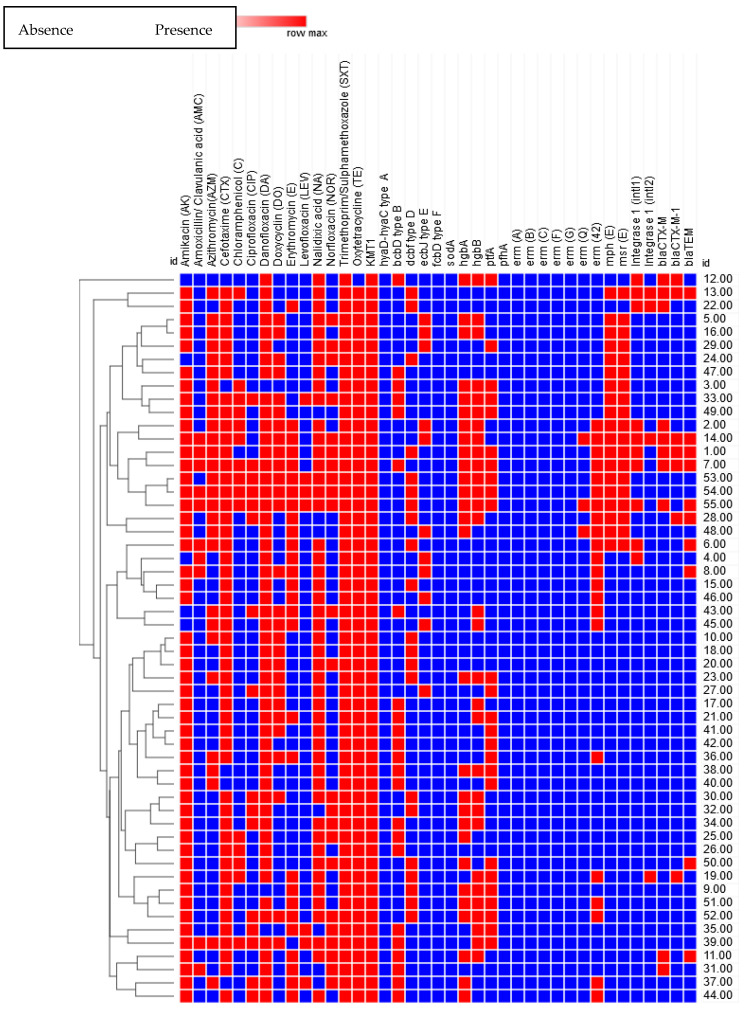
A heatmap and hierarchical clustering of the *P. multocida* isolates to 7 clusters based on their phenotypic (antimicrobial resistance), genotypic (antimicrobial resistance genes), and virulence genes expressing differences between isolates. Red represents presence and blue represents absence of phenotypic resistance, resistance genes, and virulence genes. Hierarchical clustering was performed using Wald’s method and a binary distance matrix. The numbers on the right of the heatmap refer to the isolate numbers from 1 to 55.

The relationships between virulence genes, antimicrobial resistance, resistance genes, strain, and sample sources were investigated to detect possible associations among the isolates. The phenotypic antimicrobial resistance profiles and associated genes confirmed in this study were changed to binary codes for statistical analysis. The sensitivity to a given antimicrobial agent was recorded as “0” and resistance as “1”. The presence or absence of a specific resistance gene was also scored as “1” or “0”, respectively. The Pearson correlation coefficient was calculated using the online tools at https://software.broadinstitute.org/morpheus/, accessed on 20 February 2019. Some strains exhibited high virulence, and phenotypic and genotypic antimicrobial resistance. There was a significant difference (*p* ≤ 0.05) in the impact of the strains on the virulence genes, phenotypic antimicrobial resistance, and resistance genes. Correlation matrix analyses and hierarchical clustering with heatmaps were used to detect the associations between the phenotypic and genotypic features, and the origins of the strains. There was a positive correlation between the presence of macrolide resistance genes *erm*(42), *mph*(E), and *msr*(E), and phenotypic resistance to azithromycin and erythromycin (*p* ≤ 0.05). The correlation analysis showed positive relationships between the resistance to β-lactams, especially to amoxicillin/clavulanic acid, and the presence of class 1 and 2 integrons (*intl1* and *intl2*), extended-spectrum β-lactamases (*bla*_CTX-M_ and *bla*_CTX-M-1_), and ampicillin-resistance genes (*bla*_TEM_) (Figure 6, *p* ≤ 0.05). Significant positive correlations between antibiotic resistances indicated that co-occurrence of resistance may be predominant (*p* ≤ 0.05), and it confirmed the presence of multiple drug-resistant (MDR) strains. For example, resistance to amoxicillin/clavulanic acid and cefotaxime were positively correlated with resistance to amikacin, azithromycin, danofloxacin, doxycycline, erythromycin, nalidixic acid, trimethoprim/sulfamethoxazole, and oxytetracycline. The presence of class 1 and 2 integrons (*intl1* and *intl2*), extended-spectrum β-lactamases (*bla*_CTX-M_ and *bla*_CTX-M-1_), and ampicillin-resistance gene *bla*_TEM_ was positively correlated with resistance to trimethoprim/sulfamethoxazole and oxytetracycline (*p* ≤ 0.05). Moreover, resistance to azithromycin was positively correlated with resistance to cefotaxime, danofloxacin, nalidixic acid, trimethoprim/sulfamethoxazole, and oxytetracycline. The presence of the *erm*(42), *mph*(E), and *msr*(E) genes were positively correlated with resistance to danofloxacin, nalidixic acid, trimethoprim/sulfamethoxazole, and oxytetracycline (*p* ≤ 0.05).

## 3. Discussion

*P. multocida* is a zoonotic Gram-negative bacterium responsible for many infections in domestic animals, leading to serious economic losses [23]. Along with *Mannheimia haemolytica, Mycoplasma bovis*, and *Haemophilus somnus* pneumonia, it is involved in the pathogenesis of BRD, which causes significant morbidity and mortality among cattle all over the world [1,2,14]. The isolation and biochemical identification results confirmed that *P. multocida* could be isolated from diseased and apparently healthy cattle of variable ages, ranging from weaned calves to mature cattle [24]. The results of the isolation and identification confirmed the presence of *P. multocida* in 6/48 (12.5%) of the apparently healthy cases and 44/52 (84.6%) of the visibly diseased cases. This result surpassed that of Khamesipour et al. [24] from Iran, who recovered *P. multocida* from 11.4% of pneumonic cases and 4.4% of healthy cases. The isolation of *P. multocida* from cases of human epiglottitis was confirmed by Glickman and Klein [25] from the USA, who demonstrated its presence in adults without exposure to animals, and Moyko and Ali [26], who confirmed epiglottitis in a human who was exposed to animals. As the human samples were collected from animal owners and farm workers, this could suggest that exposure to animals can enhance infection transmission to humans. The composition and structure of the capsular material found in the *P. multocida* serotypes A, D, and F are very similar to mammalian glycosaminoglycans and mainly consist of hyaluronan, heparosan, and unsulfated chondroitin, respectively [27,28,29,30]. Crude capsular antigens from *P. multocida* serogroup B were shown to contain fructose, mannose, glucose, and glucosamine [31]. There are many functions attributed to the capsules, they are as follows: (a) they are highly hydrated and protect the bacterial cell from desiccation; (b) they impart anti-phagocytic activity due to the negative charge on their surface, repelling phagocytic cells; and (c) they may impart serum resistance and aid in the evasion of the bactericidal activity of complement against capsular type A [32]. As a swifter alternative to the traditional capsular serotyping method, a multiplex PCR assay was developed. Based on the *P. multocida* characterization, detection of sequences, and analysis of the biosynthetic loci of each capsular serogroup, specific primers were developed [11].

The most prevalent capsular type among the isolates was type B (46%), which is in agreement with the findings of Ataei et al. [33], who confirmed that in tropical countries *P. multocida* serotype B:2 causes the acute disease HS in cattle and buffalo. Although capsular type D is related to infections in pigs [11], it has also been recorded in cattle infections [24]. The isolation of capsular type E from cattle was confirmed by Townsend et al. [11], who recorded its presence in African cattle. Moreover, the absence of capsular type A and F within cattle isolates was also in agreement with Townsend et al. [9]. Virulence-associated genes described for the *P. multocida* isolates include adherence and colonization factors (*ptfA*, *fimA*, *hsf-1*, *hsf-2*, *pfhA*, and *tadD*), iron-regulated and acquisition proteins (*exbB*, *exbD*, *tonB*, *hgbA*, *hgbB*, and *fur*), extracellular enzymes such as neuraminidase (*nanB* and *nanH*), hyaluronidase (*pmHAS*), superoxide dismutases (*sodA*, *sodC*, and *tbpA*), toxins (*toxA*), lipopolysaccharides (LPS), capsule proteins, and a variety of outer-membrane proteins such as protectins (*ompA*, *ompH*, *oma87*, and *plpB*) [34]. The presence of the hemoglobin-binding proteins *hgbA* and *hgbB*, involved in the iron uptake process, were confirmed in 28/55 (50.9%) and 30/55 (54.5%) of the isolates, respectively, which was lower than the findings of Khamesipour et al. [24], who showed their existence in 86.7% and 93.3% of the isolates, respectively. The presence of *hgbA* and *hgbB* in this study showed high distribution among diseased isolates, as recorded by Khamesipour et al. [24].

The *ptfA* gene, encoding type IV fimbriae, was confirmed in 25/55 (45.5%) of the isolates, which was lower than that reported by Khamesipour et al. [24], who found it in 80.0% of the screened isolates and 92.0% of the isolates from diseased cattle with pneumonic lungs. The absence of the *sodA* gene for superoxide dismutase and the *pfhA* gene encoding hemagglutinin contradicts the findings of Khamesipour et al. [24], who found these genes in 83.3% and 60.0% of the isolates, respectively. The presence of multiple virulence genes is in agreement with Khamesipour et al. [24], who reported the existence of multiple virulence genes among 30 *P. multocida* isolates obtained from pneumonic and apparently healthy slaughter cattle. The genetic profiles obtained, based on capsular type and virulence factors, were different than those reported by Khamesipour et al. [24], as a different array of virulence factors were tested; both the genetic combinations *dcbF* type D and *bcbD* type B/*hgbA*/*hgbB*/*ptfA* were the highest, with rates of 16% and 14%, respectively, while *bcbD* type B/*hgbB*/*ptfA*, *dcbF* type D/*hgbA*/*ptfA*, *dcbF* type D/*hgbB*/*ptfA*, *ecbJ* type E/*hgbA*, and *ecbJ* type E/*hgbB* were the lowest, with rates of 2% each. The capsular type and virulence factor combinations in the 55 unrelated strains comprised 17 combinations, which exhibited a discriminatory power of 0.9286, which was high enough to suggest discrimination using various genetic profiles.

Antimicrobials are often used for prophylaxis to protect potentially susceptible animals, for metaphylaxis in herds receiving new animals, and for growth promotion to improve feed efficiency [35]. The uncontrolled implementation of antimicrobials leads to a powerful selective pressure that affects the microbial community, selecting for resistance genes and resistant bacteria in the bovine digestive flora [35]. The bovine microbiota includes many harmless bacteria and opportunistic pathogens that could acquire and transmit resistance genes within the microbial community through horizontal gene transfer. Likewise, the propagation of resistance genes could influence bovine-associated human pathogens, constituting a potential public health threat [35]. Based on the results of phenotypic antimicrobial resistance, it was clear that the *P. multocida* isolates exhibited resistance to oxytetracycline, amikacin, doxycycline, erythromycin, and chloramphenicol, which surpassed the findings of Khamesipour et al. [24] from Iran, who found that cattle strains exhibited lower resistance to these antimicrobials. The resistance to amoxicillin/clavulanic acid, trimethoprim/sulfamethoxazole, ciprofloxacin, and levofloxacin was higher than that reported by El-Seedy et al. [36] from Egypt. The presence of resistance in the *P. multocida* isolates to nalidixic acid was similar to that reported by Koike et al. [37] from Hungary, while resistance to norfloxacin was higher than reports from Anwar et al. [38] from Pakistan, who found that 75% of the isolates were sensitive to norfloxacin. The resistance to azithromycin was higher than that reported by Portis et al. [14], who found that 4.6% of the *P. multocida* isolates from cattle in the USA and Canada were resistant to tulathromycin. The resistance to danofloxacin contradicts Portis et al. [14], who confirmed that *P. multocida* from cattle in the USA and Canada exhibited high susceptibility results to danofloxacin, ranging from 88.2% to 91.5% from 2000 to 2009, respectively. The high resistance in the *P. multocida* isolates to cefotaxime contradicts the results of Elshemey et al. [39] from Egypt, who confirmed that cefotaxime was the most effective antimicrobial against *P. multocida*, accompanying the foot and mouth disease virus SAT 2 FMD on bovine farms in Alexandria province, Egypt. Scarce data were found concerning the antimicrobial susceptibility profiles of *P. multocida* isolated from human epiglottitis, although Glickman and Klein [25] confirmed that an isolate from an infected 44-year-old human patient from the USA was susceptible to ceftriaxone, and Moyko and Ali [26] showed that an isolate from a 49-year-old female from the USA was susceptible to ampicillin–sulbactam.

The MAR indices of all the screened isolates ranged from 0.23 to 1. The identification of isolates with resistance to most of the antimicrobial agents widely used in veterinary practice has been documented with increasing frequency in *P. multocida* [40]. The accumulation of mutations or resistance genes usually results in multidrug resistance [41]. In *P. multocida*, San Millan et al. [42] reported multiresistance linked to the coexistence of several small plasmids encoding resistance-conferring determinants. Multiresistant, but plasmid-free, *P. multocida* isolates have been mentioned in other studies, showing that resistance genes can be associated with integrative and conjugative elements, such as ICE*Pmu1* or ICE*Pmu2* [41,43]. These elements consist of resistance gene cassettes flanked by transposase or insertion sequences, suggesting that an integration or recombination mechanism mediated by an insertion sequence can insert these resistance genes into the genome [40]. Resistance to macrolides in the family *Pasteurellaceae* resulted from a combination of at least three genes, as follows: *erm*(42), which encodes an rRNA methylase gene; *msr*(E), which encodes for a macrolide efflux pump or transporter protein; and *mph*(E), which encodes a phosphotransferase that acts through macrolide inactivation [44]. Field isolates of *P. multocida* showed high resistance to lincosamides and moderate resistance to macrolides [45]. The high distribution patterns of *erm*(42) in 22/55 (40%) of the isolates, and the equally distributed *mph*(E) and *msr*(E) genes in 19/55 (34.5%) of the isolates each, were linked to increased resistance to the macrolides erythromycin and azithromycin. This result is in agreement with those of Klima et al. [17], Desmolaize et al. [45], and Kadlec et al. [46], who found dramatic increases in the minimum inhibitory concentrations of isolates containing *erm*(42) and *msr*(E)-*mph*(E) for erythromycin, tilmicosin, and tulathromycin. The *erm*(42) was found with a high frequency among the *P. multocida* isolates. From the results of the macrolide–lincosamide–streptogramin B methylase genes, *erm*(42) encoding MLS_B_ monomethyltransferase, *mph*(E) encoding a macrolide efflux pump, and *msr*(E) encoding macrolide-inactivating phosphotransferase, 24/55 (43.6%) of the isolates harbored no resistance genes, 6/55 (10.9%) were resistant to erythromycin, 5/55 (9.1%) were resistant to azithromycin, 4/55 (7.3%) were intermediate to both antimicrobials, and 9/55 (16.4%) were sensitive to azithromycin and intermediate to erythromycin. This result of the resistant isolates harboring none of the targeted genes is in agreement with those of Ujvári et al. [40] and Rose et al. [44], who found an erythromycin-resistant *P. multocida* isolate that contained no chromosome-borne mobile genetic elements. This suggests that there is an alternative mechanism of antimicrobial resistance. The existence of many different antimicrobial resistance genes or combinations of *erm*(42) and/or *msr*(E)-*mph*(E) genes among the *P. multocida* isolates was elucidated by Rose et al. [44], who found combinations of *erm*(42) and/or *msr*(E)-*mph*(E) in 13 *P. multocida* strains. Moreover, the existence of *msr*(E) or *mph*(E) in tandem was previously confirmed by Desmolaize et al. [47]. From the results of macrolide methylase and other types of macrolide resistance genes, 12/55 (21.8%) of the isolates harbored a single resistance gene, 9/55 (16.4%) contained 2 different types, 7/55 (12.7%) harbored 3 different types, and 3/55 (5.5%) possessed 4 different types of these resistance genes. The rational interpretation of the similarity of the patterns among the isolates in harboring the same number of genes is that these antimicrobial resistance genes could be located next to transposases, and could show independent mobility within these cassettes. There is a possibility that some genes are acquired or lost in groups, while others could show individual mobility. This fact could account for the similarity and the overall diversity patterns of multidrug resistance profiles observed in the members of *Pasteurellaceae* [48,49].

The high matching between the genotype and phenotype, which constituted 23/55 (41.8%) and 22/55 (40%) for azithromycin and erythromycin, respectively, indicates the role of *erm*(42) and *msr*(E)-*mph*(E) in resistance [17,44,45]. While the existence of resistant isolates without any genes (genotype^−ve^/phenotype^+ve^) was previously reported by Ujvári et al. [40] and Rose et al. [44], suggesting alternative resistance mechanisms, the presence of sensitive strains harboring the resistance genes (genotype^+ve^/phenotype^−ve^) elucidates that these genes were inactive, as reported by Petrocchi-Rilo et al. [50].

Generally, class 1 and 2 integrons are defined by the existence of integrase genes *intI1* and *intI2* and the primary recombination sites *attI1* and *attI2*, respectively [51]. As a basis for the division of integrons into “groups”, the amino acid sequences of *intI1* integrases were used, with those carrying *intI1* classified as “class 1”, *intI2* as “class 2”, *intI3* as “class 3”, etc. The integrase genes *intI1*, *intI2*, and *intI3* were first detected in connection with mobile genetic elements, *intI4*, and others with chromosomal integrons. Both *intI1* and *intI2* encode integrases of the tyrosine recombinase family [51]. The mobility of integrons has been considered a major concern for clinical antibiotic resistance, as it has been recorded as widespread among clinical species, and is characterized as being associated with mobile DNA elements (transposons or plasmids) and genes of antibiotic resistance in addition to having a limited array size and significant heterogeneity in the *attC* site [51]. The presence of class 1 integrons among *P. multocida* from the healthy and diseased cattle was previously reported by Kong et al. [52]. Although there are no reports confirming the existence of class 2 integrons in *P. multocida*, Kehrenberg and Schwarz [53] confirmed the presence of a partially truncated class 2 integron in a *P. aerogenes* isolate. 

The resistance of *Pasteurellaceae* to β-lactam antibiotics relies on the production of β-lactamase or the existence of low-affinity penicillin-binding proteins with β-lactams [54]. Other strategies include decreased permeability of the outer membrane and systems that can effectively export β-lactams from bacterial cells, or multidrug efflux systems. These mechanisms are only rarely recorded in *Pasteurellaceae* [54], although there are five of the following β-lactamase (*bla*) genes common among *Pasteurellaceae*: *bla*_CMY-2_, *bla*_PSE-1_, *bla*_OXA-2_, *bla*_ROB-1_, and *bla*_TEM-1_. The complete *bla*_OXA-2_ gene was identified as part of ICE*Pmu1*, which was shown to be nonfunctional in *P. multocida*, but functional in *E. coli* [54]. The enzymes encoded by *bla*_CTX-M_ acquired cefotaximase resistance from a separate phylogenetic lineage to form a rapidly growing family of ESBLs with significant clinical effects [55]. Intrinsic cefotaximase chromosome-encoded genes in *Kluyvera* spp. are suggested to be the progenitors of the CTX-M family [55]. Most CTX-Ms show high activity against cefotaxime and ceftriaxone; however, some CTX-Ms exhibit enhanced catalytic efficiencies against ceftazidime, including CTX-M-15, CTX-M-16, and CTX-M-19 [55]. Records of *bla*_CTX-M_ and *bla*_CTX-M-1_ in *P. multocida* isolates from cattle and humans, either healthy or diseased, are few in Egypt, so this may be the first report confirming the existence of these ESBL genes in *P. multocida* isolates from cattle and human sources in Egypt. However, the published results from Egypt by Awad et al. [56] and Elalamy et al. [57], confirmed the presence of *bla*_ROB-1_ in 20% of the *P. multocida* isolates from diseased rabbits and 8.3% from diseased chicken, respectively. TEM-1 is able to hydrolyze penicillin and the early cephalosporins; its existence in the *P. multocida* isolates of human origin was reported by Naas et al. [58], and from cattle by Michael et al. [54], who confirmed that the presence of TEM-1 in *P. multocida* is of veterinary importance.

Several isolates demonstrated multiple drug-resistant (MDR) and extensive drug-resistant (XDR) phenotypes similar to Klima et al. [49]. Correlation analyses indicated the co-occurrence of resistance to various antimicrobials, illuminating a cause for significant concern for animal and human medicine alike. Moreover, resistance to some antimicrobials was linked with susceptibility to others; the high resistance to trimethoprim/sulfamethoxazole, oxytetracycline, danofloxacin, amikacin, cefotaxime, nalidixic acid, azithromycin, doxycycline, erythromycin, and norfloxacin were related to the low resistance to chloramphenicol, ciprofloxacin, amoxicillin/clavulanic acid, and levofloxacin. This finding is remarkable, because when discussing MDR or XDR of cattle- and human-origin *P. multocida*, it may promote the selection of alternative antimicrobials. Although there was a difference found in the screened antimicrobials against *P. multocida* by Kumar et al. [59] and our study, the interpretation of their findings proved that the susceptibility to chloramphenicol, ciprofloxacin, and amoxicillin correlated with increased resistance to amikacin, doxycycline, erythromycin, and sulfadiazine.

Correlation matrix analyses and hierarchical clustering with heatmaps were implemented to scrutinize the associations among the phenotypic and genotypic features and origin of these strains. There was a positive correlation between the macrolide resistance genes *erm*(42), *mph*(E), and *msr*(E), and the phenotypic resistance to azithromycin and erythromycin (*p* ≤ 0.05), as confirmed by Rose et al. [44] and Desmolaize et al. [45]. Furthermore, the correlation analysis showed positive relationships between the resistance to β-lactams, especially to amoxicillin/clavulanic acid, and the presence of class 1 and 2 integrons *intl1* and *intl2*, extended-spectrum β-lactamases *bla*_CTX-M_ and *bla*_CTX-M-1_, and the ampicillin-resistance gene *bla*_TEM_. Significant positive correlations of antibiotic resistances showed that the co-occurrence of resistance may be predominant (*p* ≤ 0.05), and it also confirmed the presence of MDR strains. For example, resistance to amoxicillin/clavulanic acid and cefotaxime was positively correlated with resistance to amikacin, azithromycin, danofloxacin, doxycycline, erythromycin, nalidixic acid, trimethoprim/sulfamethoxazole, and oxytetracycline. The presence of class 1 and 2 integrons *intl1* and *intl2*, extended-spectrum β-lactamase *bla*_CTX-M_ and *bla*_CTX-M-1_, and the ampicillin-resistance gene *bla*_TEM_ showed positive correlation with trimethoprim/sulfamethoxazole and oxytetracycline resistance.

## 4. Materials and Methods

### 4.1. Sampling, Isolation, and Identification

A total of 100 nasal swabs were collected from cattle of variable age, from 10-day-old calves to 24-month-old adults. There were 85 samples collected from Minufiya and 15 from Qalyubia localities in Egypt. A total of 48 samples were taken from apparently healthy cases and 52 from diseased cases, showing signs of depression, fever ≥ 41.5 °C, loss of appetite, nasal discharge, moist cough, or rapid and shallow breathing. Furthermore, 15 nasopharyngeal swabs were collected from 5 apparently healthy and 10 diseased humans suffering from signs of epiglottitis, also from Minufiya and Qalyubia. The human cases were known to be animal owners and farm workers, and written informed consent was obtained from all human participants. This research was performed according to the recommendations of the US Government for the utilization and care of vertebrate animals used in testing, research, and training. The swabs from the screened cases were transported in 10 mL of brain–heart infusion broth (Difco, New Jersey, USA). The samples were labeled, packed, cooled in an icebox, and immediately transported to the Department of Bacteriology, Mycology, and Immunology laboratory at the Faculty of Veterinary Medicine, at the University of Sadat City, and stored at 4 °C. A volume of 1 mL was transferred from the transport broth to an enrichment broth using a sterile Pasteur pipette. The enrichment broth was composed of a base of brain–heart infusion broth. Then, gentamicin (Gibco, Grand Island, NY, USA) (0.5 µg/mL), potassium tellurite (Sigma Chemical Co., St. Louis, MO, USA) (0.0125 µg/mL), amphotericin B (Sigma) (5.0 µg/mL), and defibrinated sheep blood (Remel, San Diego, CA, USA) (5%) were added. The pH was adjusted to 10.0 ± 0.1 with sterile 1 N NaOH (about 40 mL/liter). All the tubes were incubated for 16 h at 37 °C in 5–10% CO_2_. From the tubes showing turbidity, a loopful was streaked onto the selective agar medium, composed of a base of brain–heart infusion agar. The additives, concentration, and pH of the medium were adjusted in a similar manner to the enrichment broth medium [60]. The presumptive *P. multocida* colonies (3–5) were subjected to microscopical examination and biochemical identification. Briefly, *P. multocida* showed high growth on the selective medium, without hemolysis. The colonies appeared smooth, with a slightly raised center, and were mucoid and sticky in nature. No growth was observed on MacConkey agar (Difco). After staining, the *P. multocida* appeared as Gram-negative, small, cocobacilli with rounded ends. The *P. multocida* also showed positive results to the H_2_S production test, indole production test, nitrate reduction test, and catalase test, and were able to ferment glucose, sucrose, mannose, and fructose sugars. The *P. multocida* showed negative results in the methyl red, gelatin liquification, and urease tests and could not ferment lactose, maltose, or salicin [61,62].

The positive colonies were subjected to further confirmation using the Biolog GN2 and GP2 micro-plates, using the Biolog Microlog 3.70 database and software (Biolog, Hayward, CA) [63]. *P. multocida* subsp. *multocida* ATCC 12947 and *E*. *coli* ATCC 11775 strains were included as positive and negative controls, respectively.

The study design and all the experimental methods were approved by the Committee for Animal Care and Use, the Faculty of Veterinary Medicine, at the University of Sadat City, Egypt; the approval number was 2018–34. Moreover, the committee approved the implementation of human samples in this study after revision of the informed consent form.

### 4.2. Phenotypic Antimicrobial Susceptibility Profiles of P. multocida Isolates

The antimicrobial susceptibility pattern of each *P. multocida* isolate was screened against a panel of 14 antimicrobials (Oxoid, San Diego, CA, USA) (Supplementary Table S8), selected for their medical importance [64]. A volume of 1 mL of calibrated bacterial suspension (0.5 McFarland) (1.5 × 10^8^ cells/mL) was inoculated on Mueller-Hinton agar (Oxoid). *P. multocida* subsp. *multocida* ATCC12947 was used as a quality control isolate. Kirby Bauer’s disk diffusion method was utilized, and the results were interpreted according to the Clinical and Laboratory Standards Institute (CLSI) criteria [65].

### 4.3. Molecular Techniques for Capsular Classification, Detection of Virulence Genes, and Antimicrobial Resistance Genes

The genomic DNA was extracted using a QIAamp kit according to the manufacturer’s instructions (Qiagen GmbH—Germany). All 55 isolates were confirmed to be *P. multocida* using primers against *kmt1*, as described by Townsend et al. [11], encoding an integral component of the membrane. All confirmed isolates were screened for virulence genes such as *sodA*, *hgbA*, *hgbB*, *ptfA*, and *pfhA*. The presence of macrolide–lincosamide–streptogramin B methylases *erm*(A), *erm*(B), *erm*(C), *erm*(F), *erm*(G), and *erm*(Q), with *erm*(42) encoding MLS_B_ monomethyltransferase, *mph*(E) encoding a macrolide efflux pump, and *msr*(E) encoding macrolide-inactivating phosphotransferase, were also investigated in all the isolates, as was the presence of class 1 and 2 integrons *intl1* and *intl2*, extended-spectrum β-lactamases *bla*_CTX-M_ and *bla*_CTX-M-1_, and the ampicillin-resistance gene *bla*_TEM_ (Table 2). The efficiency of PCR amplification for detecting molecular capsular types, virulence genes, and antimicrobial resistance genes was confirmed by the utilization of an internal positive control from the tested *P. multocida* isolates. The positive control isolates for the molecular capsular types were isolates coded 3, 1, and 2 for capsular types B, D, and E, respectively. While the *P. multocida* isolates ATCC 12945, ATCC 12948, and NCTC 10323, with our isolate coded 3, were the positive controls for the virulence genes. Furthermore, the isolate coded 14 was the positive control for the macrolide resistance genes *erm*(Q), *erm*(42), *mph*(E), and *msr*(E). Also, this isolate coded 14 was the positive control for class 1 and 2 integrons, extended-spectrum β-lactamase, and the ampicillin-resistance genes *intl1*, *intl2*, *bla*_CTX-M_, *bla*_CTX-M-1_, and *bla*_TEM_, respectively. Moreover, the PCR for each primer was repeated three times to confirm the positive or negative result. 

The PCR protocols adopted for the confirmation, molecular capsular typing, and detection of the virulence factors of the *P. multocida* isolates were modified so as to use a simplex protocol instead of the multiplex technique described by Townsend et al. [11] and Furian et al. [64] as follows: the final volume was 10 µl, including 1 µL of 10X PCR Mg-free buffer (Invitrogen, Carlsbad, CA, USA), 2 mM MgCl_2_, 1U of Platinum Taq polymerase (Invitrogen), 0.2 mM of PCR nucleotide mix (Roche Applied Sciences, Basel, Switzerland), 1 µL of DNA template, and primers at a concentration of 3.2 µM. The PCR protocol for the amplification of resistance genes was performed as follows: the reaction volume was 25 μL, containing 12.5 μL of ready-to-use master mix, 2 μL of bacterial genomic DNA (100 ng/μL), 0.5 μL of each upstream and downstream primer (50 pmol/μL), and 9.5 μL of RNase-free water. The primers and master mix were supplied by Takara Holdings (Nojihigashi, Kusatsu, Shiga, Japan).

### 4.4. Statistical Analysis

The free online calculator for chi-square tests at https://www.socscistatistics.com/tests/chisquare2/default2.aspxaccessed, accessed on 9 February 2019 was used to detect significant differences between the recovery rates from *P. multocida*, the rates of virulence factors, virulence profile incidence, efficacies of antimicrobials, MAR indices, antimicrobial resistance gene incidence, and genotype^+^/phenotype^−^ and genotype^−^/phenotype^+^ of azithromycin and erythromycin. The phenotypic antimicrobial resistance profiles and confirmed genes were changed to numerical codes for statistical analysis. The sensitivity to antimicrobials was defined as “0” and resistance as “1”. The existence or absence of a resistance gene was defined as “1” or “0”, respectively. Heatmaps, hierarchical clustering, and Pearson correlation coefficients were calculated using the online tools at https://software.broadinstitute.org/morpheus/, accessed on 1 February 2019. The free online tools at http://insilico.ehu.es/mini_tools/discriminatory_power/index.php, accessed on 1 February 2019 were used to detect the DI of the capsular and virulence gene combinations.

## 5. Conclusions

In conclusion, *P. multocida* are widely spread among cattle and humans. We conducted a detailed screening of the virulence factors, phenotypic and genotypic antimicrobial resistance, and capsular types. The obtained isolates showed decreased resistance to chloramphenicol, ciprofloxacin, amoxicillin/clavulanic acid, and levofloxacin. Moreover, the unrationalized implementation of antimicrobials for *P. multocida* infections in cattle and humans likely aggravated the situation, and led to the emergence of multiple and extensive drug-resistant phenotypes. There were significant differences among isolates in phenotypic antimicrobial resistance, virulence genes, and resistance genes. The capsular type and virulence gene combinations observed could serve as a suitable preliminary molecular epidemiological tool with high discriminatory power. Some human isolates shared the same genotypic profiles as animal isolates, suggesting that they originated from a common source. These results will be beneficial in instating control strategies to contain *P. multocida*, and in the evaluation of related clusters, cluster expansion, and transmission dynamics.

## Figures and Tables

**Table 1:** Results of collected samples, origin, and types of cases.

Origin of Samples		Types of Cases			
		Apparently Healthy		Diseased	
		No.	%	No.	%
Minufiya	Cattle (85)	40	40/100 (40)	45	45/100 (45)
	Human (7)	2	2/15 (13.3)	5	5/15 (33.3)
Qalyubia	Cattle (15)	8	8/100 (8)	7	7/100 (7)
	Human (8)	3	3/15 (20)	5	5/15 (33.3)
Total collected samples	Cattle (100)	48	48/100 (48)	52	52/100 (52)
	Human (15)	5	5/15 (33.3)	10	10/15 (66.7)
Positive isolation	Cattle (50)	6 (5 from Minufiya and 1 from Qalyubia)	6/48 (12.5)	44 (40 from Minufiya and 4 from Qalyubia)	44/52 (84.6)
	Human (5)	0	0/15 (0.0)	5 from Qalyubia	5/15 (33.3)

**Table 2:** Primer sequences, anticipated amplicon size, and amplification conditions.

Name	Amplicon Size (bp)	Cycle Number	Annealing Temperature (°C) and Time (Seconds)	Primer Sequence	Purpose	Reference
*kmt1*	460	30	55 °C, 30 s	KMT1T7-ATCCGCTATTTACCCAGTGG KMT1SP6-GCTGTAAACGAACTCGCCAC	Confirmation of isolates using primer targeting an integral component of membrane	[11]
*hyaD-hyaC*	1044	30	55 °C, 30 s	CAPA-TGCCAAAATCGCAGTCAG CAPA-TTGCCATCATTGTCAGTG	Molecular capsular typing	
*bcbD*	760	30	55 °C, 30 s	CAPB-CATTTATCCAAGCTCCACC CAPB-GCCCGAGAGTTTCAATCC		
*dcbF*	657	30	55 °C, 30 s	CAPD-TTACAAAAGAAAGACTAGGAGCCC CAPD-CATCTACCCACTCAACCATATCAG		
*ecbJ*	511	30	55 °C, 30 s	CAPE-TCCGCAGAAAATTATTGACTC CAPE-GCTTGCTGCTTGATTTTGTC		
*fcbD*	851	30	55 °C, 30 s	CAPF-AATCGGAGAACGCAGAAATCAG CAPF-TTCCGCCGTCAATTACTCTG		
*sodA*	361	25	55 °C, 30 s	F:TACCAGAATTAGGCTACGC R:GAAACGGGTTGCTGCCGCT	Superoxide dismutase	[66]
*hgbA*	419	25	55 °C, 30 s	F:TGGCGGATAGTCATCAAG R:CCAAAGAACCACTACCCA	Iron uptake	
*hgbB*	788	25	55 °C, 30 s	F:ACCGCGTTGGAATTATGATTG R:CATTGAGTACGGCTTGACAT	Iron uptake	
*ptfA*	488	25	55 °C, 30 s	F:TGTGGAATTCAGCATTTTAGTGTGTC R:TCATGAATTCTTATGCGCAAAATCCTGCTGG	Type IV fimbriae	
*pfhA*	275	25	55 °C, 30 s	F:AGCTGATCAAGTGGTGAAC RTGGTACATTGGTGAATGCTG	Hemagglutinin	
*erm*(A)	157	40	63 °C, 30 s	F:AGTCAGGCTAAATATAGCTATC R:CAAGAACAATCAATACAGAGTCTAC	Macrolide–lincosamide–streptogramin B methylases	[67]
*erm*(B)	191	40	65 °C, 30 s	F:GGTTGCTCTTGCACACTCAAG R:CAGTTGACGATATTCTCGATTG		
*erm*(C)	293	40	63 °C, 30 s	F:AATCGTGGAATACGGGTTTGC R:CGTCAATTCCTG CATGTTTTAAGG		
*erm*(F)	424	40	65 °C, 30 s	F:TCTGGGAGGTTCCATTGTCC R:TTCAGGGACAACTTCCAG C		
*erm*(G)	255	40	63 °C, 30 s	F:GTGAGGTAACTCGTAATAAGCTG R:CCTCTGCCATTAACAGCAATG		
*erm*(Q)	154	35	68 °C, 30 s	F:CACCAACTGATATGTGGCTAG R:CTAGGCATGGGATGGAAGTC		
*erm*(42)	173	25	68 °C, 30 s	F:TGCACCATCTTACAAGGAGT R:CATGCCTGTCTTCAAGGTTT	MLS_B_ monomethyltransferase	[43]
*mph*(E)	271	25	68 °C, 30 s	F:ATGCCCAGCATATAAATCGC R:ATATGGACAAAGATAGCCCG	Macrolide efflux pump	
*msr*(E)	395	25	68 °C, 30 s	F:TATAGCGACTTTAGCGCCAA R:GCCGTAGAATATGAGCTGAT	Macrolide-inactivating phosphotransferase	
*intl1*	280	33	64 °C, 30 s	F:CCTCCCGCACGATGATC R:TCCACGCATCGTCAGGC	Detect class 1 and 2 integrons	[68]
*intl2*	300	33	64 °C, 30 s	F:GCAAACGCAAGCATTCATTA R:ACGGATATGCGACAAAAAGG		
*bla*_CTX-M_	500	35	55 °C, 1 min	F:TTTGCGATGTGCAGTACCAGTAA R:CTCCGCTGCCGGTTTTATC	Detect extended-spectrum β-lactamase	
*bla*_CTX-M-1_	415	35	55 °C, 1 min	F:AAAAATCACTGCGCCAGTTC R:AGCTTATTCATCGCCACGTT		
*bla*_TEM_	800	30	55 °C, 1 min	F:CCGTGTCGCCCTTATTCC R:AGGCACCTATCTCAGCGA	Ampicillin-resistance gene	

## Data Availability

The data represented in this study are available in the article or Supplementary Materials.

## Supplementary Materials

The following are available online at https://www.mdpi.com/article/10.3390/antibiotics10050480/s1, Table S1. Detailed results of isolation of *P. multocida* on specific media in relation to origin, age, and signs of cases. Table S2. Results of molecular confirmation of *P. multocida* isolates, detection of capsular types, and virulence genes. Table S3. Results of the most prevalent genotypes. Table S4. Results of antimicrobial susceptibility testing and multiple antimicrobial resistance indices of tested *P. multocida* isolates. Table S5. Results of distribution patterns of macrolide resistance genes. Table S6. Numbers and percentages of Azithromycin and Erythromycin resistant *P. multocida* isolates with discrepancies between genotype-phenotype. Table S7. Results of class 1 and 2 integrons, extended-spectrum β-lactamase, ampicillin-resistance gene. Table S8. The list, categorization and prioritization of antimicrobials classified as critically important in human and veterinary medicine.

## References

[1] Zhao G., He H., Wang H. (2019). Use of a recombinase polymerase amplification commercial kit for rapid visual detection of *Pasteurella multocida*. BMC Vet. Res..

[2] Stanford K., Zaheer R., Klima C., McAllister T., Peters D., Niu Y.D., Ralston B. (2020). Antimicrobial resistance in members of the bacterial bovine respiratory disease complex isolated from lung tissue of cattle mortalities managed with or without the use of antimicrobials. Microorganisms.

[3] Arashima Y., Kubo N., Iwasaki Y., Okuyama K., Kumasaka K., Tsuchiya T., Kawano K., Ootsuka M., Saitoh F., Namikawa K. (1990). Human respiratory tract infection by *Pasteurella multocida* subsp. *multocida* presumably derived from the cat. Kansenshogaku Zasshi..

[4] Guo Y., McMullen C., Timsit E., Hallewell J., Orsel K., van der Meer F., Yan S., Alexander T.W. (2020). Genetic relatedness and antimicrobial resistance in respiratory bacteria from beef calves sampled from spring processing to 40 days after feedlot entry. Vet. Microbiol..

[5] Annas S., Zamri-Saad M., Jesse F.F., Zunita Z. (2014). New sites of localisation of *Pasteurella multocida* B:2 in buffalo surviving experimental haemorrhagic septicaemia. BMC Vet. Res..

[6] Al-Maary K.S., Dawoud T.M., Mubarak A.S., Hessain A.M., Galal H.M., Kabli S.A., Mohamed M.I. (2017). Molecular characterization of the capsular antigens of *Pasteurella multocida* isolates using multiplex PCR. Saudi J. Biol. Sci..

[7] St Michael F., Harper M., Parnas H., John M., Stupak J., Vinogradov E., Adler B., Boyce J.D., Cox A.D. (2009). Structural and genetic basis for the serological differentiation of *Pasteurella multocida* Heddleston serotypes 2 and 5. J. Bacteriol..

[8] Carter G.R. (1961). A new serological type of *Pasteurella multocida* from central Africa. Vet. Rec..

[9] Rimler R.B., Rhoades K.R. (1987). Serogroup F. A new capsule serogroup of *Pasteurella multocida*. J. Clin. Microbiol..

[10] Peng Z., Liang W., Wu B. (2016). Molecular typing methods for *Pasteurella multocida*—A review. Acta Microbiol. Sin..

[11] Townsend K.M., Boyce J.D., Chung J.Y., Frost A.J., Adler B. (2001). Genetic organization of *Pasteurella multocida* cap Loci and development of a multiplex capsular PCR typing system. J. Clin. Microbiol..

[12] Harper M., John M., Turni C., Edmunds M., St Michael F., Adler B., Blackall P.J., Cox A.D., Boyce J.D. (2015). Development of a rapid multiplex PCR assay to genotype *Pasteurella multocida* strains by use of the lipopolysaccharide outer core biosynthesis locus. J. Clin. Microbiol..

[13] Peng Z., Liang W., Wang F., Xu Z., Xie Z., Lian Z., Hua L., Zhou R., Chen H., Wu B. (2018). Genetic and phylogenetic characteristics of *Pasteurella multocida* isolates from different host species. Front. Microbiol..

[14] Portis E., Lindeman C., Johansen L., Stoltman G. (2012). A ten-year (2000–2009) study of antimicrobial susceptibility of bacteria that cause bovine respiratory disease complex—*Mannheimia haemolytica*, *Pasteurella multocida*, and *Histophilus somni*—In the United States and Canada. J. Vet. Diagn. Investig..

[15] DeDonder K.D., Apley M.D. (2015). A literature review of antimicrobial resistance in pathogens associated with bovine respiratory disease. Anim. Health Res. Rev..

[16] Anholt R.M., Klima C., Allan N., Matheson-Bird H., Schatz C., Ajilkumar P., Otto S.J.G., Peters D., Schmid K., Olson M. (2017). Antimicrobial susceptibility of bacteria that cause bovine respiratory disease complex in Alberta Canada. Front. Vet. Sci..

[17] Klima C.L., Zaheer R., Cook S.R., Booker C.W., Hendrick S., Alexander T.W., McAllister T.A. (2014). Pathogens of bovine respiratory disease in north american feedlots conferring multidrug resistance via integrative conjugative elements. J. Clin. Microbiol..

[18] Feng Y., Qi W., Wang X., Wang L., Li X., Luo J., Zhang S., Li H. (2016). Genetic characterization of antimicrobial resistance in *Staphylococcus aureus* isolated from bovine mastitis cases in northwest China. J. Integr. Agric..

[19] EFSA (2011). Scientific Opinion on the public health risks of bacterial strains producing extendedspectrum β-lactamases and/or AmpC β-lactamases in food and food-producing animals. EFSA J..

[20] Michael G.B., Freitag C., Wendlandt S., Eidam C., Feßler A.T., Lopes G.V., Kadlec K., Schwarz S. (2015). Emerging issues in antimicrobial resistance of bacteria from food-producing animals. Future Microbiol..

[21] Bush K., Jacoby G.A. (2010). Updated functional classification of beta-lactamases. Antimicrob. Agents Chemother..

[22] Ur Rahman S., Ali T., Ali I., Khan N.A., Han B., Gao J. (2018). The growing genetic and functional diversity of extended spectrum beta-lactamases. BioMed Res. Int..

[23] Steen J.A., Steen J.A., Harrison P., Seemann T., Wilkie I., Harper M., Adler B., Boyce J.D. (2010). Fis is essential for capsule production in *Pasteurella multocida* and regulates expression of other important virulence factors. PLOS Pathog..

[24] Khamesipour F., Momtaz H., Azhdary Mamoreh M. (2014). Occurrence of virulence factors and antimicrobial resistance in *Pasteurella multocida* strains isolated from slaughter cattle in Iran. Front. Microbiol..

[25] Glickman M., Klein R.S. (1997). Acute Epiglottitis due to *Pasteurella multocida* in an Adult without Animal Exposure. Emerg. Infect. Dis..

[26] Moyko A., Ali N.J., Dubosh N.M., Wong M.L. (2017). *Pasteurella multocida* Epiglottitis. Clin. Pract. Cases Emerg. Med..

[27] Pandit K.K., Smith J.E. (1993). Capsular hyaluronic acid in *Pasteurella multocida* type A and its counterpart in type D. Res. Vet. Sci..

[28] Rimler R.B., Rhoades K.R. (1994). Hyaluronidase and chondroitinase activity of *Pasteurella multocida* serotype B:2 involved in hemorrhagic septicaemia. Vet. Rec..

[29] DeAngelis P.L. (1996). Enzymological characterization of the *Pasteurella multocida* hyaluronic acid synthase. Biochemistry.

[30] DeAngelis P.L., Padgett-McCue A.J. (2000). Identification and molecular cloning of a chondroitin synthase from *Pasteurella multocida* type F. J. Biol. Chem..

[31] Knox K.W., Bain R.V.S. (1960). The antigens of *Pasteurella multocida* type I. Immunology.

[32] Boyce J.D., Chung J.Y., Adler B. (2000). *Pasteurella multocida* capsule: Composition, function and genetics. J. Biotechnol..

[33] Ataei Kachooei S., Ranjbar M.M., Ataei Kachooei S. (2017). Evaluation of *Pasteurella multocida* serotype B:2 resistance to immune serum and complement system. Vet. Res. Forum.

[34] Katoch S., Sharma M., Patil R.D., Kumar S., Verma S. (2014). In vitro and in vivo pathogenicity studies of *Pasteurella multocida* strains harbouring different ompA. Vet. Res. Commun..

[35] Cameron A., McAllister T.A. (2016). Antimicrobial usage and resistance in beef production. J. Anim. Sci. Biotechnol..

[36] El-Seedy F.R., Abed A.H., Hassan H.M., Nabih A.M., Khalifa E., Salem S.E. (2019). Antimicrobial and immunological studies on *Pasteurella multocida* and *Mannheimia haemolytica* recovered from calves affected with respiratory manifestations. JVMR.

[37] Koike S., Inoue K., Yoneyama S., Ichikawa Y., Tajima K. (2009). Antimicrobial susceptibility of respiratory bacterial pathogen isolated from cattle for the past 16 years in Tochigi Prefecture. JVMA.

[38] Anwar M.A.H., Rahman S.U., Ahmad R. (2000). Antibiotic Sensitivity of *Pasteurella multocida* isolated from cattle and buffaloes. Pak. J. Biol. Sci..

[39] Elshemey T.M., Abd-Elrahman A.H. (2013). Hemorrhagic septicemia outbreak as a consequence to SAT2 FMD infection in buffalo and cattle in Alexandria province, Egypt. Life Sci. J..

[40] Ujvári B., Makrai L., Magyar T. (2018). Characterisation of a multiresistant *Pasteurella multocida* strain isolated from cattle. Acta Vet. Hung..

[41] Michael G.B., Kadlec K., Sweeney M.T., Brzuszkiewicz E., Liesegang H., Daniel R., Murray R.W., Watts J.L., Schwarz S. (2012). ICE*Pmu1*, an integrative conjugative element (ICE) of *Pasteurella multocida*: Analysis of the regions that comprise 12 antimicrobial resistance genes. J. Antimicrob. Chemother..

[42] San Millan A., Escudero J.A., Gutierrez B., Hidalgo L., Garcia N., Llagostera M., Dominguez L., Gonzalez-Zorn B. (2009). Multiresistance in *Pasteurella multocida* is mediated by coexistence of small plasmids. Antimicrob. Agents Chemother..

[43] Moustafa A.M., Seemann T., Gladman S., Adler B., Harper M., Boyce J.D., Bennett M.D. (2015). Comparative genomic analysis of Asian haemorrhagic septicaemia-associated strains of *Pasteurella multocida* identifies more than 90 haemorrhagic septicaemia-specific genes. PLoS ONE.

[44] Rose S., Desmolaize B., Jaju P., Wilhelm C., Warrass R., Douthwaite S. (2012). Multiplex PCR to identify macrolide resistance determinants in *Mannheimia haemolytica* and *Pasteurella multocida*. Antimicrob. Agents Chemother..

[45] Desmolaize B., Rose S., Warrass R., Douthwaite S. (2011). A novel Erm monomethyltransferase in antibiotic-resistant isolates of *Mannheimia haemolytica* and *Pasteurella multocida*. Mol. Microbiol..

[46] Kadlec K., Brenner Michael G., Sweeney M.T., Brzuszkiewicz E., Liesegang H., Daniel R., Watts J.L., Schwarz S. (2011). Molecular basis of macrolide, triamilide, and lincosamide resistance in *Pasteurella multocida* from bovine respiratory disease. Antimicrob. Agents Chemother..

[47] Desmolaize B., Rose S., Wilhelm C., Warrass R., Douthwaite S. (2011). Combinations of macrolide resistance determinants in field isolates of *Mannheimia haemolytica* and *Pasteurella multocida*. Antimicrob. Agents Chemother..

[48] Klima C.L., Cook S.R., Zaheer R., Laing C., Gannon V.P., Xu Y., Rasmussen J., Potter A., Hendrick S., Alexander T.W. (2016). Comparative genomic analysis of *Mannheimia haemolytica* from bovine sources. PLoS ONE.

[49] Klima C.L., Holman D.B., Cook S.R., Conrad C.C., Ralston B.J., Allan N., Anholt R.M., Niu Y.D., Stanford K., Hannon S.J. (2020). Multidrug resistance in *Pasteurellaceae* associated with bovine respiratory disease mortalities in north America from 2011 to 2016. Front. Microbiol..

[50] Petrocchi-Rilo M., Gutiérrez-Martín C.B., Pérez-Fernández E., Vilaró A., Fraile L., Martínez-Martínez S. (2020). Antimicrobial resistance genes in porcine *Pasteurella multocida* are not associated with its antimicrobial susceptibility pattern. Antibiotics.

[51] Deng Y., Bao X., Ji L., Chen L., Liu J., Miao J., Chen D., Bian H., Li Y., Yu G. (2015). Resistance integrons: Class 1, 2 and 3 integrons. Ann. Clin. Microbiol. Antimicrob..

[52] Kong L.C., Wang Z., Wang Y.M., Dong W.L., Jia B.Y., Gao D., Jiang X.Y., Ma H.X. (2019). Antimicrobial susceptibility and molecular typing of *Pasteurella multocida* isolated from six provinces in China. Trop. Anim. Health Prod..

[53] Kehrenberg C., Schwarz S. (2011). Trimethoprim resistance in a porcine *Pasteurella aerogenes* isolate is based on a *dfrA1* gene cassette located in a partially truncated class 2 integron. J. Antimicrob. Chemother..

[54] Michael G.B., Bossé J.T., Schwarz S. (2018). Antimicrobial resistance in *Pasteurellaceae* of veterinary origin. Microbiol. Spectr..

[55] Zhao W.H., Hu Z.Q. (2013). Epidemiology and genetics of CTX-M extended-spectrum β-lactamases in Gram-negative bacteria. Crit. Rev. Microbiol..

[56] Awad N.F.S., Abd El-Hamid M.I. (2019). Coexistence of virulence and antibiotic resistance genes in *Pasteurella multocida* isolated from diseased rabbits. Zagazig Vet. J..

[57] Elalamy R.A., Tartor Y.H., Ammar A.M., Eldesouky I.E., Esawy A.E.I. (2020). Molecular characterization of extensively drug-resistant *Pasteurella multocida* isolated from apparently healthy and diseased chickens in Egypt. Pak. Vet. J..

[58] Naas T., Benaoudia F., Lebrun L., Nordmann P. (2001). Molecular identification of TEM-1 β-lactamase in a *Pasteurella multocida* isolate of human origin. Eur. J. Clin. Microbiol. Infect. Dis..

[59] Kumar P., Singh V.P., Agrawal R.K., Singh S. (2009). Identification of *Pasteurella multocida* isolates of ruminant origin using polymerase chain reaction and their antibiogram study. Trop. Anim. Health Prod..

[60] Moore M.K., Cicnjak-Chubbs L., Gates R.J. (1994). A new selective enrichment procedure for isolating *Pasteurella multocida* from avian and environmental samples. Avian Dis..

[61] Kozarev A., Mamadudian B.A. (1988). Biochemical properties of *Pasteurella multocida* strains from ruminants. Vet. Sibrika.

[62] Ashraf A., Tariq H., Shah S., Nadeem S., Manzoor I., Ali S., Ijaz A., Gailani S., Mehboob S. (2011). Characterization of *Pasteurella multocida* strains isolated from cattle and buffaloes in Karachi, Pakistan. Afr. J. Microbiol. Res..

[63] Sellyei B., Wehmann E., Makrai L., Magyar T. (2011). Evaluation of the Biolog system for the identification of certain closely related *Pasteurella* species. Diagn. Microbiol. Infect. Dis..

[64] WHO (World Health Organization) (2017). Critically Important Antimicrobials for Human Medicine—5th Revision.

[65] CLSI (2014). Performance Standards for Antimicrobial Susceptibility Testing.

[66] Furian T.Q., Borges K.A., Rocha S.L.S., Rodrigues E.E., Nascimento V.P., Salle C.T.P., Moraes H.L.S. (2013). Detetion of virulence-associated genes of *Pasteurella multocida* isolated from cases of fowl cholera by multiplex-PCR. Pesq. Vet. Bras..

[67] Elsayed M.S.A.E., Roshdey T., Salah A., Tarabees R., Younis G., Eldeep D. (2019). Phenotypic and genotype methods for identification of slime layer production, efflux pump activity, and antimicrobial resistance genes as potential causes of the antimicrobial resistance of some mastitis pathogens from farms in Menoufia, Egypt. Mol. Biol. Rep..

[68] Raphael E., Wong L.K., Riley L.W. (2011). Extended-spectrum beta-lactamase gene sequences in Gram-negative saprophytes on retail organic and nonorganic spinach. Appl. Environ. Microbiol..

