# Supplementary material for: Virulence Determinants and Antimicrobial Profiles of Pasteurella multocida Isolated from Cattle and Humans in Egypt
Source: Antibiotics (Basel). 2021 Apr 22;10(5):480. doi: 10.3390/antibiotics10050480 (PMC8143532; doi:10.3390/antibiotics10050480)
Supplement: Supplementary file 1 [file antibiotics-10-00480-s001.zip › antibiotics-1132962-supplementary.pdf]

antibiotics

Article

Virulence Determinants and Antimicrobial Profiles of *Pasteurella multocida* Isolated from Cattle and Humans in Egypt

Mohamed Sabry Abd Elraheam Elsayed [1,*], Samah Mahmoud Eldsouky [2], Tamer Roshdy [3], Lamia Said [4], Nahed Thabet [4], Tamer Allam [4], A.B. Abeer Mohammed [5], Ghada M. Nasr [6], Mohamed S. M. Basiouny [7], Behairy A. Akl [8], Maha M. Nader [8], Al Shaimaa Hasan [9] and Ahmed Salah [3]

1 Department of Bacteriology, Mycology, and Immunology, Faculty of Veterinary Medicine, University of Sadat City, Minufiya 32897, Egypt; mohamed.sabry@vet.usc.edu.eg
2 Department of Otolaryngology and Head and Neck Surgery, Faculty of Medicine, Benha University, Benha city, Qalyubia 13511, Egypt; samahmahmoed@yahoo.com
3 Department of Molecular Biology, Genetic Engineering and Biotechnology Research Institute, University of Sadat City, Sadat City, Minufiya, Egypt; tmr_gebri@yahoo.com (T.R.); ahmed.salah@gebri.usc.edu.eg (A.S.)
4 Department of Clinical Pathology, Faculty of Veterinary Medicine, University of Sadat City, Egypt; lamiaasaid@yahoo.com (L.S.); nahedthabet@yahoo.com (N.T.); tamerallam@yahoo.com (T.A.)
5 Department of Microbial Biotechnology, Genetic Engineering and Biotechnology Research Institute, University of Sadat City, Egypt; beromicro@gmail.com
6 Department of Molecular Diagnostics, Genetic Engineering and Biotechnology Research Institute, University of Sadat City, Egypt; nasr_mi@yahoo.com
7 Faculty of Biotechnology, Badr University, Cairo; Mohamed-Salah@buc.edu.eg
8 Microbiology Department, Faculty of Agriculture, Zagazig University, Egypt; Beharyakl2005@yahoo.com; mahanaderdiab@gmail.com
9 Department of Medical Pharmacology, Faculty of Medicine, South Valley University, Qena, Egypt; DRelshimaa.hassan@med.svu.edu.eg
* Correspondence: mohammedelsayed529@yahoo.com; Tel.: +02001012692475

Citation: Elsayed, M.S.A.E.; Eldsouky, S.M.; Said, L.; Roshdy, T.; Said, L.; Thabet, N.; Allam, T.; Mohammed, A.; Naser, G.M.; Basiouny, M.S.M.; Akl, B.A.; Nader, M.M.; Hasan, A.S. and Salah, A. Virulence Determinants and Antimicrobial Profiles of *Pasteurella multocida* Isolated from Cattle and Humans in Egypt. *Antibiotics* **2021**, *10*, 480. https://doi.org/10.3390/antibiotics10050480

Academic Editor: Michael Calcutt

Received: 17 February 2021
Accepted: 15 April 2021
Published: 22 April 2021

Publisher's Note: MDPI stays neutral with regard to jurisdictional claims in published maps and institutional affiliations.

Copyright: © 2021 by the authors. Submitted for possible open access publication under the terms and conditions of the Creative Commons Attribution (CC BY) license (http://creativecommons.org/licenses/by/4.0/).

Table S1. Detailed results of isolation of *P. multocida* on specific media in relation to origin, age, and signs of cases.

Case number	Origin	Age (months for animals and year for human)	Types of cases		Isolation
			Diseased	Apparently healthy	
1	Minufiya	18	+	-	+
2	Minufiya	24	+	-	+
3	Minufiya	24	+	-	+
4	Minufiya	12	+	-	+
5	Minufiya (Sadat City)	18	+	-	+
6	Minufiya	24	+	-	+
7	Minufiya	6	-	+	+
8	Qalyubia	12	+	-	+
9	Qalyubia	24	+	-	+
10	Minufiya	18	+	-	+
11	Qalyubia	12	-	+	+
12	Minufiya	6	+	-	+
13	Minufiya	24	+	-	+
14	Minufiya	24	+	-	+
15	Minufiya	12	+	-	+
16	Qalyubia	24	+	-	+
17	Minufiya	24	+	-	+
18	Minufiya (Sadat City)	18	+	-	+
19	Minufiya	12	-	+	+
20	Qalyubia	24	+	-	+
21	Minufiya	24	+	-	+
22	Minufiya	3	+	-	+
23	Minufiya	24	+	-	+
24	Minufiya	18	+	-	+
25	Minufiya	24	+	-	+
26	Minufiya (Sadat City)	24	+	-	+
27	Minufiya	12	+	-	+
28	Minufiya (Sadat City)	12	+	-	+
29	Qalyubia	24	+	-	+
30	Minufiya	6	+	-	+
31	Minufiya (Sadat City)	24	+	-	+
32	Minufiya	18	+	-	+
33	Minufiya	24	-	+	+
34	Minufiya	12	+	-	+
35	Minufiya (Sadat City)	10 days	+	-	+
36	Minufiya	18	+	-	+
37	Minufiya (Sadat City)	24	+	-	+
38	Minufiya (Sadat City)	24	+	-	+
39	Minufiya	18	-	+	+
40	Minufiya (Sadat City)	6	+	-	+

41	Minufiya	24	+	-	+
42	Qalyubia	12	+	-	+
43	Minufiya	12	+	-	+
44	Minufiya	24	-	+	+
45	Minufiya (Sadat City)	12	+	-	+
46	Minufiya	2	+	-	+
47	Minufiya (Sadat City)	12	+	-	+
48	Minufiya (Sadat City)	12	+	-	+
49	Minufiya (Sadat City)	12	+	-	+
50	Minufiya (Sadat City)	24	+	-	+
51	Qalyubia (Human)	45	+	-	+
52	Qalyubia (Human)	40	+	-	+
53	Qalyubia (Human)	49	+	-	+
54	Minufiya (Human)	44	+	-	+
55	Minufiya (Human)	46	+	-	+
Total			49/55 (89.1%)	6/55 (10.9%)	55/115 (47.8%)

Table S2. Results of molecular confirmation of *P. multocida* isolates, detection of capsular types, and virulence genes.

Isolate number	State of case	Capsular types					Virulence factors					No of virulence genes per each isolate
		hyaD-hyaC type A	*bcbD* type B	*dcbf* type D	*ecbJ* type E	*fcbD* type F	*sodA*	*hgbA*	*hgbB*	*ptfA*	*pfhA*	
3	Diseased	-	+	-	-	-	-	+	+	+	-	3
7	Apparently healthy	-	+	-	-	-	-	+	+	+	-	3
12	Diseased	-	+	-	-	-	-	+	+	+	-	3
33	Apparently healthy	-	+	-	-	-	-	+	+	+	-	3
38	Diseased	-	+	-	-	-	-	+	+	+	-	3
49	Diseased	-	+	-	-	-	-	+	+	+	-	3
11	Apparently healthy	-	+	-	-	-	-	+	+	-	-	2
34	Diseased	-	+	-	-	-	-	+	+	-	-	2
21	Diseased	-	+	-	-	-	-	-	+	+	-	2
35	Diseased	-	+	-	-	-	-	-	+	+	-	2
39	Apparently healthy	-	+	-	-	-	-	-	+	+	-	2
25	Diseased	-	+	-	-	-	-	+	-	-	-	1
37	Diseased	-	+	-	-	-	-	+	-	-	-	1
44	Apparently healthy	-	+	-	-	-	-	+	-	-	-	1
17	Diseased	-	+	-	-	-	-	-	+	-	-	1

43	Diseased	-	+	-	-	-	-	-	+	-	-	1
36	Diseased	-	+	-	-	-	-	-	-	+	-	1
40	Diseased	-	+	-	-	-	-	-	-	+	-	1
41	Diseased	-	+	-	-	-	-	-	-	+	-	1
42	Diseased	-	+	-	-	-	-	-	-	+	-	1
26	Diseased	-	+	-	-	-	-	-	-	-	-	0
31	Diseased	-	+	-	-	-	-	-	-	-	-	0
47	Diseased	-	+	-	-	-	-	-	-	-	-	0
1	Diseased	-	-	+	-	-	-	+	+	+	-	3
9	Diseased	-	-	+	-	-	-	+	+	+	-	3
51	Diseased (Human)	-	-	+	-	-	-	+	+	+	-	3
52	Diseased (Human)	-	-	+	-	-	-	+	+	+	-	3
53	Diseased (Human)	-	-	+	-	-	-	+	+	+	-	3
54	Diseased (Human)	-	-	+	-	-	-	+	+	+	-	3
55	Diseased (Human)	-	-	+	-	-	-	+	+	+	-	3
23	Diseased	-	-	+	-	-	-	+	+	+	-	3
28	Diseased	-	-	+	-	-	-	+	+	-	-	2
30	Diseased	-	-	+	-	-	-	+	+	-	-	2
32	Diseased	-	-	+	-	-	-	+	+	-	-	2
19	Apparently healthy	-	-	+	-	-	-	-	+	+	-	2
50	Diseased	-	-	+	-	-	-	+	-	+	-	2
6	Diseased	-	-	+	-	-	-	-	-	-	-	0
10	Diseased	-	-	+	-	-	-	-	-	-	-	0
13	Diseased	-	-	+	-	-	-	-	-	-	-	0
15	Diseased	-	-	+	-	-	-	-	-	-	-	0
18	Diseased	-	-	+	-	-	-	-	-	-	-	0
20	Diseased	-	-	+	-	-	-	-	-	-	-	0
22	Diseased	-	-	+	-	-	-	-	-	-	-	0
24	Diseased	-	-	+	-	-	-	-	-	-	-	0
2	Diseased	-	-	-	+	-	-	+	+	-	-	2
5	Diseased	-	-	-	+	-	-	+	+	-	-	2
14	Diseased	-	-	-	+	-	-	+	+	-	-	2
16	Diseased	-	-	-	+	-	-	+	+	-	-	2
48	Diseased	-	-	-	+	-	-	+	-	-	-	1
45	Diseased	-	-	-	+	-	-	-	+	-	-	1
27	Diseased	-	-	-	+	-	-	-	-	+	-	1
29	Diseased	-	-	-	+	-	-	-	-	+	-	1
4	Diseased	-	-	-	+	-	-	-	-	-	-	0
8	Diseased	-	-	-	+	-	-	-	-	-	-	0
46	Diseased	-	-	-	+	-	-	-	-	-	-	0
Total		0/55	23/55	21/55	11/55	0/55	0/55	28/55	30/55	25/55	0/55	

(0.0%)	(41.8%)	(38.1%)	(20%)	(0.0%)	(0.0%)	(50.9%)	(54.5%)	(45.5%)	(0.0%)

Table S3. Results of the most prevalent genotypes.

No.	Genotype	Isolate number	Percentage
1	*bcbD* type B	26<br>31<br>47	3/55 (5.5%)
2	*bcbD* type B, *hgbA*	25<br>37<br>44	3/55 (5.5%)
3	*bcbD* type B, *hgbB*	17<br>43	2/55 (3.6%)
4	*bcbD* type B, *hgbA*, *hgbB*	11<br>34<br>38	3/55 (5.5%)
5	*bcbD* type B, *hgbB*, *ptfA*	21	1/55 (1.8%)
6	*bcbD* type B, *ptfA*	36<br>40<br>41<br>42	4/55 (7.3%)
7	*bcbD* type B, *hgbA*, *hgbB*, *ptfA*	3<br>7<br>12<br>33<br>35<br>39<br>49	7/55 (12.7%)
8	*dcbf* type D	6<br>10<br>13<br>15<br>18<br>20<br>22<br>24	8/55 (14.5%)
9	*dcbf* type D, *hgbA*, *hgbB*	28<br>30<br>23	3/55 (5.5%)
10	*dcbf* type D, *hgbA*, *ptfA*	50	1/55 (1.8%)
11	*dcbf* type D, *hgbB*, *ptfA*	19	1/55 (1.8%)
12	*dcbf* type D, *hgbA*, *hgbB*, *ptfA*	1<br>9<br>23<br>51<br>52<br>53<br>54<br>55	8/55 (14.5%)
13	*ecbJ* type E	4	3/55 (5.5%)

		8	
		46	
14	*ecbJ* type E, *hgbA*	48	1/55 (1.8%)
15	*ecbJ* type E, *hgbB*	45	1/55 (1.8%)
16	*ecbJ* type E, *hgbA*, *hgbB*	2<br>5<br>14<br>16	4/55 (7.3%)
17	*ecbJ* type E, *ptfA*	27<br>29	2/55 (3.6%)

Table S4. Results of antimicrobial susceptibility testing and multiple antimicrobial resistance indices of tested *P. multocida* isolates.

Isolate no.	Antimicrobials													
	**Amikacin (AK)**	**Amoxicillin/ clavulinic acid**	**Azithromycin (AZM)**	**Cefotaxime (CTX)**	**Chloramphenicol (C)**	**Ciprofloxacin (CIP)**	**Danofloxacin (DA)**	**Doxycycline (DO)**	**Erythromycin(E)**	**Levofloxacin (LEV)**	**Nalidixic acid (NA)**	**Norfloxacin (NOR)**	**Trimethoprim/ Sulphamethoxazole**	**Oxytetracycline (TE)**	MAR index
1 Diseased	R	R	R	R	S	I	R	R	R	S	R	R	R	R	0.85
2 Diseased	R	I	R	R	R	I	R	R	R	S	R	I	R	R	0.69
3 Diseased	R	S	R	S	R	S	I	S	I	S	R	S	R	R	0.46
4 Diseased	S	R	S	R	S	S	R	S	R	S	R	S	R	R	0.46
5 Diseased	R	S	R	R	S	I	R	R	I	I	R	R	R	R	0.69
6 Diseased	R	R	R	R	S	S	R	S	R	S	R	S	R	R	0.62
7Apparently healthy	R	R	R	R	R	R	R	R	R	S	R	R	R	R	0.92
8 Diseased	R	R	I	R	S	I	R	R	R	I	R	I	R	R	0.64
9 Diseased	R	S	S	R	S	S	I	S	R	S	R	S	R	R	0.38
10 Diseased	R	S	R	R	S	I	R	R	I	S	R	S	R	R	0.62
11 Apparently healthy	R	I	I	R	S	I	R	S	R	S	R	R	R	R	0.62
12 Diseased	S	I	I	S	S	S	R	S	I	S	R	S	R	I	0.23
13 Diseased	R	I	R	R	R	I	R	S	I	S	R	I	R	R	0.62

14 Diseased	R	R	R	R	R	I	R	R	R	S	R	R	R	R	0.85
15 Diseased	R	S	I	R	I	I	R	S	R	S	R	I	R	R	0.46
16 Diseased	R	S	R	R	S	I	R	R	I	S	R	I	R	R	0.64
17 Diseased	R	S	I	R	I	S	R	R	I	I	R	I	R	R	0.54
18 Diseased	R	S	S	R	S	I	R	R	I	S	R	I	R	R	0.54
19 Apparently healthy	R	I	I	R	R	I	R	S	R	S	R	I	R	R	0.54
20 Diseased	R	S	S	R	S	I	R	R	I	S	R	R	R	R	0.62
21 Diseased	R	S	S	R	S	I	R	R	R	I	R	S	R	R	0.54
22 Diseased	R	I	I	R	S	I	R	I	R	S	R	S	R	R	0.46
23 Diseased	R	S	R	R	S	S	R	R	S	S	R	S	R	R	0.62
24 Diseased	I	S	R	R	S	I	R	R	I	S	R	R	R	R	0.62
25 Diseased	R	S	I	R	R	I	R	S	I	S	R	R	R	R	0.62
26 Diseased	R	S	I	R	R	I	R	S	I	S	R	I	R	R	0.54
27 Diseased	R	S	S	R	S	R	R	R	I	S	R	I	R	R	0.62
28 Diseased	R	I	R	R	S	R	R	I	R	S	I	S	R	R	0.54
29 Diseased	R	S	R	R	S	I	R	S	I	S	R	R	R	R	0.62
30 Diseased	R	S	S	R	S	R	R	R	I	S	R	R	R	R	0.69
31 Diseased	R	R	S	R	I	S	R	S	R	S	I	R	R	R	0.54
32 Diseased	R	S	S	R	I	R	R	I	I	S	I	R	R	R	0.54
33 Apparently healthy	R	S	R	R	R	R	R	R	I	R	R	R	R	R	0.92
34 Diseased	R	S	S	R	S	R	R	S	I	I	R	R	R	R	0.62
35 Diseased	R	S	I	R	I	R	I	S	R	R	I	R	R	R	0.54
36 Diseased	R	S	R	R	S	S	R	R	R	S	R	S	R	R	0.62
37 Diseased	R	S	R	S	S	R	R	S	R	R	I	R	R	R	0.62

38 Diseased	R	S	R	S	S	S	R	S	I	S	R	S	R	R	0.46
39 Apparently healthy	R	R	R	R	R	R	R	R	I	R	R	R	R	R	1
40 Diseased	R	S	R	S	S	I	R	I	I	S	R	I	R	R	0.46
41 Diseased	R	S	S	R	S	I	R	R	I	S	R	I	R	R	0.54
42 Diseased	R	S	S	R	S	S	R	S	I	S	R	S	R	R	0.46
43 Diseased	S	S	R	R	I	R	R	R	R	I	R	R	R	R	0.69
44 Apparently healthy	R	S	S	R	S	R	R	S	R	S	R	I	R	R	0.54
45 Diseased	S	S	R	R	S	I	R	R	R	S	R	S	R	R	0.54
46 Diseased	R	S	I	R	S	I	R	I	R	S	R	I	R	R	0.46
47 Diseased	R	S	R	R	S	I	R	R	S	S	R	I	R	R	0.62
48 Diseased	R	S	R	R	S	I	R	I	R	S	I	S	R	R	0.46
49 Diseased	R	S	R	R	R	I	R	R	I	S	I	S	R	R	0.62
50 Diseased	R	I	S	R	R	S	R	S	I	S	R	R	R	R	0.62
51 Diseased (Human)	R	S	S	R	S	S	R	S	R	S	R	S	R	R	0.5
52 Diseased (Human)	R	S	S	R	S	R	R	R	R	S	R	R	R	R	0.72
53 Diseased (Human)	R	S	R	R	R	R	R	R	R	R	R	R	R	R	0.93
54 Diseased (Human)	R	R	R	R	R	R	R	R	R	R	R	R	R	R	1
55 Diseased (Human)	R	R	R	R	R	R	R	R	R	R	R	R	R	R	1
Sensitive (S)	4/55 (7.3%)	37/55 (67.3%)	16/55 (29.09%)	5/55 (9.09%)	34/55 (61.8%)	13/55 (23.6%)	0/55 (0.0%)	21/55 (38.2%)	2/55 (3.6%)	42/55 (76.4%)	0/55 (0.0%)	17/55 (30.9%)	0/55 (0.0%)	0/55 (0.0%)	
Intermediate (I)	1/55 (1.8%)	8/55 (14.5%)	11/55 (20%)	0/55 (0.0%)	6/55 (10.9%)	26/55 (47.3%)	3/55 (5.5%)	6/55 (10.9%)	25/55 (45.5%)	6/55 (10.9%)	7/55 (12.7%)	15/55 (27.3%)	0/55 (0.0%)	1/55 (1.8%)	

Resistant (R)	50/55 (90.9%)	10/55 (18.2%)	28/55 (50.9%)	50/55 (90.9%)	15/55 (27.3%)	16/55 (29.09%)	52/55 (94.5%)	28/55 (50.9%)	28/55 (50.9%)	7/55 (12.7%)	48/55 (87.3%)	23/55 (41.8%)	55/55 (100%)	54/50 (98.2%)

R: resistant, S: sensitive, I: intermediate.

Table S5. Results of distribution patterns of macrolide resistance genes.

Isolate number	Azithromycin (AZM)	Erythromycin (E)	*erm (A)*	*erm (B)*	*erm (C)*	*erm (F)*	*erm (G)*	*erm (Q)*	*erm (42)*	*mph (E)*	*msr (E)*	No. of copies per each isolate
1 Diseased	R	R	-	-	-	-	-	-	+	+	+	3
2 Diseased	R	R	-	-	-	-	-	-	+	+	+	
6 Diseased	R	R	-	-	-	-	-	-	+	+	+	
7 pparently healthy	R	R	-	-	-	-	-	-	+	+	+	
28 Diseased	R	R	-	-	-	-	-	-	+	+	+	
53Diseased (Human)	R	R	-	-	-	-	-	-	+	+	+	
54Diseased (Human)	R	R	-	-	-	-	-	-	+	+	+	
36 Diseased	R	R	-	-	-	-	-	-	+	-	-	1
37 Diseased	R	R	-	-	-	-	-	-	+	-	-	
43 Diseased	R	R	-	-	-	-	-	-	+	-	-	
45 Diseased	R	R	-	-	-	-	-	-	+	-	-	
14 Diseased	R	R	-	-	-	-	-	+	+	+	+	4
48 Diseased	R	R	-	-	-	-	-	+	+	+	+	
55Diseased (Human)	R	R	-	-	-	-	-	+	+	+	+	
4 Diseased	S	R	-	-	-	-	-	-	+	-	-	1
44 Apparently healthy	S	R	-	-	-	-	-	-	+	-	-	
9 Diseased	S	R	-	-	-	-	-	-	-	-	-	0
21 Diseased	S	R	-	-	-	-	-	-	-	-	-	
31 Diseased	S	R	-	-	-	-	-	-	-	-	-	
51Diseased (Human)	S	R	-	-	-	-	-	-	+	-	-	1
52Diseased (Human)	S	R	-	-	-	-	-	-	+	-	-	
8 Diseased	I	R	-	-	-	-	-	-	+	-	-	

15 Diseased	I	R	-	-	-	-	-	-	+	-	-	
19 pparently healthy	I	R	-	-	-	-	-	-	+	-	-	
11Apparently healthy	I	R	-	-	-	-	-	-	-	-	-	0
22 Diseased	I	R	-	-	-	-	-	-	-	-	-	
35 Diseased	I	R	-	-	-	-	-	-	-	-	-	
46 Diseased	I	R	-	-	-	-	-	-	+	-	-	1
3 Diseased	R	I	-	-	-	-	-	-	-	+	+	2
5 Diseased	R	I	-	-	-	-	-	-	-	+	+	
13 Diseased	R	I	-	-	-	-	-	-	-	+	+	
16 Diseased	R	I	-	-	-	-	-	-	-	+	+	
24 Diseased	R	I	-	-	-	-	-	-	-	+	+	
29 Diseased	R	I	-	-	-	-	-	-	-	+	+	
33 Apparently healthy	R	I	-	-	-	-	-	-	-	+	+	
10 Diseased	R	I	-	-	-	-	-	-	-	-	-	0
38 Diseased	R	I	-	-	-	-	-	-	-	-	-	
39 Apparently healthy	R	I	-	-	-	-	-	-	-	-	-	
40 Diseased	R	I	-	-	-	-	-	-	-	-	-	
49 Diseased	R	I	-	-	-	-	-	-	-	+	+	2
18 Diseased	S	I	-	-	-	-	-	-	-	-	-	0
20 Diseased	S	I	-	-	-	-	-	-	-	-	-	
27 Diseased	S	I	-	-	-	-	-	-	-	-	-	
30 Diseased	S	I	-	-	-	-	-	-	-	-	-	
32 Diseased	S	I	-	-	-	-	-	-	-	-	-	
34 Diseased	S	I	-	-	-	-	-	-	-	-	-	
41 Diseased	S	I	-	-	-	-	-	-	-	-	-	
42 Diseased	S	I	-	-	-	-	-	-	-	-	-	
50 Diseased	S	I	-	-	-	-	-	-	-	-	-	
23 Diseased	R	S	-	-	-	-	-	-	-	-	-	
47 Diseased	R	S	-	-	-	-	-	-	-	+	+	2
12 Diseased	I	I	-	-	-	-	-	-	-	-	-	0
17 Diseased	I	I	-	-	-	-	-	-	-	-	-	
25 Diseased	I	I	-	-	-	-	-	-	-	-	-	
26 Diseased	I	I	-	-	-	-	-	-	-	-	-	
Total			0/55 (0.0%)	0/55 (0.0%)	0/55 (0.0%)	0/55 (0.0%)	0/55 (0.0%)	3/55 (5.5%)	22/55 (40%)	19/55 (34.5%)	19/55 (34.5%)	

R: resistant, S: sensitive, I: intermediate.

0: no *erm* gene copies, 1: one *erm* copy, 2: two *erm* gene copies, 3: three *erm* copies, 4: four *erm* copies.

Table S6. Numbers and percentages of Azithromycin and Erythromycin resistant *P*. multocida isolates with discrepancies between genotype-phenotype.

Category	No. (%) of isolates	
	Azithromycin	Erythromycin
Genotype^{+ve}/Phenotype^{-ve}	8/55 (14.5)^{a}	1/55 (1.8)^{a}
Genotype^{+ve}/Phenotype^{+ve} (Match no discrepancy)	23/55 (41.8)	22/55 (40)
Genotype^{-ve}/Phenotype^{+ve}	4 (7.2)^{b}	6/55 (10.9)^{b}
Total	35/55 (63.6)	29/55 (52.7)

Negative (-ve), positive (+ve).

Comparing a and b of Azithromycin *P* = 0.1021.

Comparing a and b of Erythromycin *P* <0.05.

Table S7. Results of class 1 and 2 integrons, extended-spectrum β-lactamase, ampicillin-resistance gene.

Isolate number	Amoxicillin/ clavulinic acid (AMC)	Integrase 1 (*intl*1)	Integrase 2 (*intl*2)	*bla*CTX-M	*bla*CTX-M-1	*bla*TEM	No. of copies per each isolate
1 Diseased	R	+	-	+	+	+	4
2 Diseased	I	+	-	+	-	-	2
3 Diseased	S	-	-	-	-	-	0
4 Diseased	R	+	-	-	-	-	1
5 Diseased	S	-	-	-	-	-	0
6 Diseased	R	+	-	-	-	+	2
7Apparently healthy	R	+	-	+	+	+	4
8 Diseased	R	-	-	-	-	+	1
9 Diseased	S	-	-	-	-	-	0
10 Diseased	S	-	-	-	-	-	0
11 Apparently healthy	I	-	-	+	-	+	2
12 Diseased	I	+	-	+	+	-	3
13 Diseased	I	+	+	+	+	+	5
14 Diseased	R	+	+	+	+	+	5
15 Diseased	S	-	-	-	-	-	0
16 Diseased	S	-	-	-	-	-	0
17 Diseased	S	-	-	-	-	-	0
18 Diseased	S	-	-	-	-	-	0

19 Apparently healthy	I	-	+	-	+	-	2
20 Diseased	S	-	-	-	-	-	0
21 Diseased	S	-	-	-	-	-	0
22 Diseased	I	+	+	+	-	-	3
23 Diseased	S	-	-	-	-	-	0
24 Diseased	S	-	-	-	-	-	0
25 Diseased	S	-	-	-	-	-	0
26 Diseased	S	-	-	-	-	-	0
27 Diseased	S	-	-	-	-	-	0
28 Diseased	I	-	-	-	+	+	2
29 Diseased	S	-	-	-	-	-	0
30 Diseased	S	-	-	-	-	-	0
31 Diseased	R	-	-	+	-	-	1
32 Diseased	S	-	-	-	-	-	0
33Apparently healthy	S	-	-	-	-	-	0
34 Diseased	S	-	-	-	-	-	0
35 Diseased	S	-	-	-	-	-	0
36 Diseased	S	-	-	-	-	-	0
37 Diseased	S	-	-	-	-	-	0
38 Diseased	S	-	-	-	-	-	0
39Apparently healthy	R	-	-	-	-	-	0
40 Diseased	S	-	-	-	-	-	0
41 Diseased	S	-	-	-	-	-	0
42 Diseased	S	-	-	-	-	-	0
43 Diseased	S	-	-	-	-	-	0
44Apparently healthy	S	-	-	-	-	-	0
45 Diseased	S	-	-	-	-	-	0
46 Diseased	S	-	-	-	-	-	0
47 Diseased	S	-	-	-	-	-	0
48 Diseased	S	-	-	-	-	-	0
49 Diseased	S	-	-	-	-	-	0
50 Diseased	I	-	-	-	-	+	1
51Diseased (Human)	S	-	-	-	-	-	0
52Diseased (Human)	S	-	-	-	-	-	0
53Diseased (Human)	S	-	-	-	-	-	0
54Diseased (Human)	R	-	-	-	-	-	0
55Diseased (Human)	R	+	-	+	-	+	3
Total		10/55 (18.2%)	4/55 (7.2%)	10/55 (18.2%)	7/55 (12.2%)	10/55 (18.2%)	

Table S8. The list, categorization and prioritization of antimicrobials classified as critically important in human and veterinary medicine.

Antimicrobial agents	Disc concentration	Antimicrobial class	Medical importance of antimicrobial	Prioritization criterion
Amikacin	30µg	Aminoglycosides	High priority critically important	P2 and P3
Amoxicillin/ clavulanic acid	30µg	Penicillins	Highest priority critically important	P2 and P3
Cefotaxime	30µg	Cephalosporins	Highest priority critically important	P1 and P2
Azithromycin	15µg	Macrolides and ketolides	Highest priority critically important	P1 and P2
Chloramphenicol	15µg	Amphenicols	Highly important	P2
Ciprofloxacin	5µg	Quinolones and fluoroquinolones	Highest priority critically important	P1 and P2
Danofloxacin	5µg	Quinolones and fluoroquinolones	Highest priority critically important	P1 and P2
Levofloxacin	5µg	Quinolones and fluoroquinolones	Highest priority critically important	P1 and P2
Naldixic acid	30µg	Quinolones and fluoroquinolones	Highest priority critically important	P1 and P2
Norfloxacin	10µg	Quinolones and fluoroquinolones	Highest priority critically important	P1 and P2
Doxycycline	20µg	Tetracyclines	Highest priority critically important	P1
Oxytetracyclin	20µg	Tetracyclines	Highest priority critically important	P1
Erythromycin	20µg	Macrolides and ketolides	Highest priority critically important	P1, P2 and P3

Trimethoprim/ Sulphamethoxazole	23.75μg/1.25μg	Sulfonamides, dihydrofolate reductase inhibitors and combinations	Highly important	P2
